# New species based on the biological species concept within the complex of *Lariophagus distinguendus* (Hymenoptera, Chalcidoidea, Pteromalidae), a parasitoid of household pests

**DOI:** 10.1002/ece3.10524

**Published:** 2023-09-13

**Authors:** Marie Pollmann, Denise Kuhn, Christian König, Irmela Homolka, Sina Paschke, Ronja Reinisch, Anna Schmidt, Noa Schwabe, Justus Weber, Yuval Gottlieb, Johannes Luitpold Maria Steidle

**Affiliations:** ^1^ Department of Chemical Ecology 190t, Institute of Biology University of Hohenheim Stuttgart Germany; ^2^ Department of Entomology 360c, Institute of Phytomedicine University of Hohenheim Stuttgart Germany; ^3^ Akademie für Natur‐ und Umweltschutz Baden‐Württemberg Stuttgart Germany; ^4^ Plant Evolutionary Biology 190b, Institute of Biology University of Hohenheim Stuttgart Germany; ^5^ Robert H. Smith Faculty of Agriculture, Food and Environment, Koret School of Veterinary Medicine Hebrew University of Jerusalem Rehovot Israel; ^6^ KomBioTa – Center of Biodiversity and Integrative Taxonomy University of Hohenheim Stuttgart Germany

**Keywords:** cryptic diversity, parasitoid wasps, reproductive isolation, species delimitation, turbo‐taxonomy

## Abstract

The pteromalid parasitoid *Lariophagus distinguendus* (Foerster) belongs to the Hymenoptera, a megadiverse insect order with high cryptic diversity. It attacks stored product pest beetles in human storage facilities. Recently, it has been shown to consist of two separate species. To further study its cryptic diversity, strains were collected to compare their relatedness using barcoding and nuclear genes. Nuclear genes identified two clusters which agree with the known two species, whereas the barcode fragment determined an additional third Clade. Total reproductive isolation (RI) according to the biological species concept (BSC) was investigated in crossing experiments within and between clusters using representative strains. Sexual isolation exists between all studied pairs, increasing from slight to strong with genetic distance. Postzygotic barriers mostly affected hybrid males, pointing to Haldane's rule. Hybrid females were only affected by unidirectional *Spiroplasma*‐induced cytoplasmic incompatibility and behavioural sterility, each in one specific strain combination. RI was virtually absent between strains separated by up to 2.8% COI difference, but strong or complete in three pairs from one Clade each, separated by at least 7.2%. Apparently, each of these clusters represents one separate species according to the BSC, highlighting cryptic diversity in direct vicinity to humans. In addition, these results challenge the recent ‘turbo‐taxonomy’ practice of using 2% COI differences to delimitate species, especially within parasitic Hymenoptera. The gradual increase in number and strength of reproductive barriers between strains with increasing genetic distance also sheds light on the emergence of barriers during the speciation process in *L. distinguendus*.

## Graphical abstract

We studied cryptic diversity of a hymenopterous parasitoid occurring in human households. We identified three separate species based on the biological species concept, which were not recovered using conventional thresholds in COI and nuclear markers. Our study also sheds light on the emergence of barriers during the speciation process in Hymenoptera.

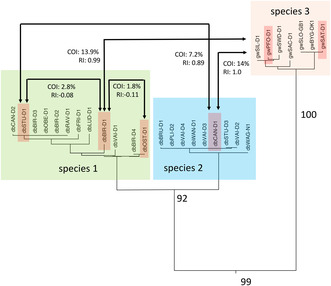

## 1 INTRODUCTION

*Lariophagus distinguendus* (Foerster) (Pteromalidae) is a pteromalid wasp that belongs to the superfamily Chalcidoidea within the Hymenoptera, which is an extremely speciose, if not the most speciose animal order (Forbes et al., 2018). The Chalcidoidea comprise more than 20,000 described species (Noyes, 2019), and new species are discovered on a regular basis (Aguiar et al., 2013). This is partly due to a high abundance of cryptic species and the existence of numerous species complexes (e.g. Chesters et al., 2012; Desneux et al., 2009; Fusu, 2017; Heimpel et al., 1997; Heraty et al., 2007; König, Zundel, et al., 2019). For the Chalcidoidea, a high speciation rate is hypothesized to be caused by a high abundance of sib‐mating, which quickly restricts gene flow between populations, and by the haplodiploid mode of sex determination, which causes rapid elimination of deleterious alleles in the haploid males and a rapid selection of favourable gene combinations (Askew, 1968; Malec et al., 2021).

*L. distinguendus* is a parasitoid of coleopteran larvae from at least 17 beetle species in six families (Niedermayer et al., 2016). Most of its hosts occur in grain stores and pantries of households and are pests on dry stored plant products like grain, corn and pasta. Therefore, *L. distinguendus* can be used as biological control agent against its hosts (Niedermayer et al., 2016; Niedermayer & Steidle, 2013). Previous studies established the existence of at least two distinct cryptic species within *L. distinguendus*, which are almost indistinguishable morphologically (König, Zundel, et al., 2019; Wendt et al., 2014). These two species, which remain undescribed so far, were provisionally named GW species and DB species after their preferred hosts, the granary weevil *Sitophilus granarius* (Coleoptera: Curculionidae) L. and the drugstore beetle *Stegobium paniceum* (Coleoptera: Ptinidae) L., respectively. Barriers between the species are formed by differences in host and habitat preferences, different numbers of chromosomes, sexual and postzygotic isolation, as well as endosymbiont‐induced cytoplasmic incompatibility (CI) caused by the bacterium *Spiroplasma* (Gokhman et al., 2019; König, Krimmer, et al., 2015; König, Seeger, et al., 2015; König, Zundel, et al., 2019; König, Paschke, et al., 2019; Pollmann et al., 2022). The bacterium *Wolbachia*, which is known to induce CI as reproductive barrier between *Nasonia* species (Bordenstein et al., 2001; Breeuwer & Werren, 1990), was also found in the GW species, but did not cause reproductive isolation (König, Zundel, et al., 2019). Recently, we collected a larger number of *L. distinguendus* strains in the area of Stuttgart in Southern Germany, a largely urban area. Phylogenetic analyses of some of these strains based on non‐barcode parts of the mitochondrial cytochrome C oxidase subunit I (COI) gene indicate that all belong to the already established DB species and share the same chromosome numbers (König, Paschke, et al., 2019). However, this analysis also revealed well‐supported sub‐Clades, which could indicate that the DB species might be in fact a complex of two or three distinct species. So far, no analysis of the barcode region of the mitochondrial COI gene sequences as well as of nuclear genes has been conducted for the majority of these new strains. In addition, there are no studies on reproductive isolation for these strains. Thus, it is unclear if more cryptic species exist within the DB species.

Here, we investigated the hidden diversity in the *L. distinguendus* species complex by addressing the following two questions: Are there more distinct species among the *L. distinguendus* strains traceable (1) by genetic divergence and (2) based on the biological species concept? For species delimitation, we reconstructed phylogenetic trees based on the barcoding COI gene as well as five nuclear genes (Hajibabaei et al., 2006; Hebert et al., 2004; Smith et al., 2005, 2006; Ward et al., 2005) and examined potential species status according to the biological species concept (BSC) (Coyne & Orr, 2004; Mayr, 1969). To that end, we investigated pre‐ and postzygotic isolating barriers, that is, barriers occurring before and after fertilization, in particular sexual isolation, hybrid viability, sterility and reduced fertility (Coyne & Orr, 2004) in strains representative of the different genetically determined clusters using crossing experiments.

Our results enable a comparison between the heavily discussed approach of ‘turbo‐taxonomy’ (Butcher et al., 2012) for species delimitation based on a 2% divergence in COI (Meierotto et al., 2019; Sharkey et al., 2021) and the traditional BSC (Coyne & Orr, 2004; Mayr, 1969). In addition, because the divergence in COI in the studied strain pairs ranges along a gradient from 1.7% to 14%, our study also allows to draw conclusions on the potential emergence of reproductive barriers during the speciation process in *L. distinguendus*.

## 2 MATERIALS AND METHODS

### 2.1 Studied insects

All studied individuals of *L. distinguendus* were reared at the Department of Chemical Ecology of the University of Hohenheim. Almost all wasp strains were collected in Germany, except for three strains originating from Great Britain, The Netherlands and Denmark respectively. Strains were named after their respective collection site and host species, that is, either larvae of drugstore beetles or granary weevils (see Table 1 for details). Both hosts were obtained from the Julius Kühn‐Institut in Berlin. For the rearing of drugstore beetles, honey jars (diameter 12 cm, height 16 cm) with ventilated lids containing 80 g of koi pellets (Hikari Friend, Kamihata Fish Industry Group, Kyorin Corporation, Japan) were inoculated with approximately 1 g of beetles. Larvae suitable for parasitization were obtained after around 6 weeks at 26–27°C, 45% r.h. and a natural L:D cycle determined by the light from outdoors. For granary weevil cultures, weevils were placed in honey jars containing 200 mL of wheat grains (*Tritium aestivum* L.) for oviposition and removed after 1 week. The wheat was moistened (1 mL water/40 g wheat grains). To obtain 4‐week‐old larvae, which are suitable as hosts, these cultures were kept at 25°C and a L:D cycle of 16:8 for 3 weeks, and at 20°C and a natural L:D cycle for 1 week. Then, they were transferred to 15°C. DB strains were reared on drugstore beetle larvae in honey jars with koi pellets, except for the strains BIR and WAG, which were reared on drugstore beetle larvae infesting wheat grains in Petri dishes. GW strains were reared on granary weevil larvae in Petri dishes containing wheat grains. All wasp strains were maintained by transferring newly hatched wasps onto new host substrate in regular intervals and kept at 26°C, a relative humidity of 45%–50% and a natural L:D cycle.

**TABLE 1:** *Lariophagus distinguendus* strains with host species and the localities the collection sites belong to.

Strain	Short name	Host	Locality of the collection site	Used^c^
dbBIR‐D1	BIR	*St. p*.^a^	Stuttgart‐Birkach, Baden‐Württemberg, Germany	+
dbBIR‐D2	−	*St. p*.	Stuttgart‐Birkach, Baden‐Württemberg, Germany	−
dbBIR‐D3	−	*St. p*.	Stuttgart‐Birkach, Baden‐Württemberg, Germany	−
dbBIR‐D4	−	*St. p*.	Stuttgart‐Birkach, Baden‐Württemberg, Germany	−
dbBRU‐D1	−	*St. p*.	Bruchsal, Baden‐Württemberg, Germany	−
gwBYG‐DK1		*S. g*.^b^	Bygholm, Horsens Kommune, Region Midtjylland, Denmark	−
dbCAN‐D1	CAN	*St. p*.	Stuttgart‐Bad Cannstatt, Baden‐Württemberg, Germany	+
dbCAN‐D2	−	*St. p*.	Stuttgart‐Bad Cannstatt, Baden‐Württemberg, Germany	−
dbFRI‐D1	−	*St. p*.	Fritzlar, Hesse, Germany	−
dbLUD‐D1	−	*St. p*.	Ludwigsburg, Baden‐Württemberg, Germany	−
dbOBE‐D1	−	*St. p*.	Stuttgart‐Obertürkheim, Baden‐Württemberg, Germany	−
dbOST‐D1	OST	*St. p*.	Ostfildern, Baden‐Württemberg, Germany	+
gwPFO‐D1	PFO	*S. g*.	Pforzheim, Baden‐Württemberg, Germany	+
dbPLI‐D2	−	*St. p*.	Stuttgart‐Plieningen, Baden‐Württemberg, Germany	−
dbRAV‐D1	RAV	*St. p*.	Ravensburg, Baden‐Württemberg, Germany	−
gwSAC‐D1	−	*S. g*.	Saxony, Germany	−
gwSAT‐D1	SAT	*S. g*.	Satrup, Schleswig‐Holstein, Germany	−
gwSIL‐D1	−	*S. g*.	Stuttgart‐Sillenbuch, Baden‐Württemberg, Germany	−
gwSLO‐GB1	−	*S. g*.	Slough, Berkshire, Great Britain	−
dbSTU‐D1	STU	*St. p*.	Stuttgart‐Bad Cannstatt, Baden‐Württemberg, Germany	+
dbSTU‐D3	−	*St. p*.	Stuttgart West, Baden‐Württemberg, Germany	−
gwSWD‐D1	−	*S. g*.	Schwieberdingen, Baden‐Württemberg, Germany	−
dbVAI‐D1	−	*St. p*.	Stuttgart‐Vaihingen, Baden‐Württemberg, Germany	−
dbVAI‐D2	−	*St. p*.	Stuttgart‐Vaihingen, Baden‐Württemberg, Germany	−
dbVAI‐D3	−	*St. p*.	Stuttgart‐Vaihingen, Baden‐Württemberg, Germany	−
dbVAI‐D4	−	*St. p*.	Stuttgart‐Vaihingen, Baden‐Württemberg, Germany	−
dbWAG‐N1	−	*St. p*.	Wageningen, Gelderland The Netherlands	−
dbWAN‐D1	−	*St. p*.	Stuttgart‐Wangen, Baden‐Württemberg, Germany	−

*Note*: Strains with a preference for drugstore beetles are labelled with the prefix db, whereas the prefix gw designates strains preferring granary weevils. Consecutively numbered strains named for the same localities were collected at different collection sites within these localities.

^a^ *St. paniceum*.

^b^ *S. granarius*.

^c^ + − used in crossing experiments.

Wasps used for crossing experiments were isolated prior to eclosion to ensure their virginity. Therefore, single infested wheat grains were separated in 1.5 mL Eppendorf tubes and wasps developing in koi pellets were removed as pupae by dissection and isolated in 1.5 mL Eppendorf tubes.

### 2.2 Antibiotic treatment

To remove endosymbionts, namely *Wolbachia* in the GW strains (König, Zundel, et al., 2019) and *Spiroplasma* in dbSTU‐D1 (Pollmann et al., 2022), which could impair the results of the crossing experiments and phylogenetic analyses as described below, tetracycline‐treated lines were generated for the strains gwPFO‐D1, gwSAT‐D1, gwBYG‐D1, gwSAC‐D1, gwSLO‐GB1, dbBIR‐D1 and dbSTU‐D1. Wasps were placed in Petri dishes containing filter paper and a piece of cotton wool soaked with a solution of 10 mg tetracycline (Sigma‐Aldrich Chemie GmbH, Taufkirchen, Germany) and 1 g sucrose per 10 mL water before being moved to their respective host substrate for oviposition after 24 h. After three generations with antibiotic treatment, the elimination of endosymbionts was confirmed by polymerase chain reaction (PCR) as described below.

### 2.3 DNA extraction, gene amplification and sequencing

Genomic DNA (gDNA) was extracted from individual wasps using the nexttec 1‐step tissue & cells kit (nexttec Biotechnologie GmbH, Hilgertshausen, Germany) by following the manufacturer's instructions. All gDNA samples were stored at −20°C until further processing. To amplify the five nuclear genes, the following primer combinations were used: Carbamoyl phosphate synthase domain of the conserved ATPase domain (CAD): CAD f (5′‐CAG TTC GAT GAA GAG CGT AGG‐3′)/CAD r (5′‐ATA GAC ACC CGA ACC TTT GAA GA‐3′) (Klopfstein et al., 2013), parts of the internal transcribed spacer 2 (ITS2): ITS2 f (5′‐TGT GAA CTG CAG GAC ACA TG‐3′)/ITS2 r (5′‐ATG CTT AAA TTT AGG GGG T‐3′) (Quicke et al., 2006), LOC100123206 (LOC1): HOG4652_10 f (5′‐GGW TTT GGY TTT ATT CGT TG‐3′)/HOG4652_10 r (5′‐YTC TTT ATT YCG YTT YAC TTG‐3′), LOC100123909 (LOC2): HOG5134_01 f (5′‐AGT AAA ATG GGT YTW ATG TC‐3′)/HOG5134_01 r (5′‐STR TTC CAR TTW ACT CCR TA‐3′) and LOC100117339 (LOC3): (HOG5592_08 f (5′‐YAA YGA GGA CCA ATC GAG AT‐3′)/HOG5592_08 r (5′‐GCA TWA CRA TAG ATC TYG CTT CTC‐3′) without sequencing tails (Hartig et al., 2012). To amplify the COI region of the mitochondrial region, the primer combination LCO1490 (5′‐GGT CAA CAA ATC ATA AAG ATA TTG G‐3′)/HCO2198 (5′‐TAA ACT TCA GGG TGA CCA AAA AAT CA‐3′) (Folmer et al., 1994) was used. Both CAD and ITS2 were amplified at 95°C for 5 min followed by 40 cycles of 94°C for 1 min, 55°C for 1 min and 72°C for 1.5 min and finally at 72°C for 10 min. For amplification with the COI and W‐Spec primers, PCR conditions consisted of an initial denaturation at 95°C for 2 min, 32 cycles of 94°C for 30 s, 49°C for 45 s and 72°C for 1 min, and a final elongation step at 72°C for 1 min. LOC3 was amplified using 94°C for 4 min, 35 cycles of 94°C for 1 min, 49°C for 1 min and 72°C for 1.5 min, followed by 72°C for 1 min. Touchdown PCRs consisting of 4 min at 94°C for initial elongation, two cycles of 94°C for 1 min, 52°C for 1 min and 72°C for 1.5 min, two cycles of 94°C for 1 min, 50°C for 1 min and 72°C for 1.5 min, 36 cycles of 94°C for 1 min, 48°C for 1 min and 72°C for 1.5 min and a final elongation step at 72°C for 5 min, were conducted for LOC1 and LOC2. PCRs were conducted either with 12.5 μL of ROTI®Pol TaqS Red‐Mix (2×) (Carl Roth GmbH + Co. KG), 1 μL of each primer and 9.5 μL of double distilled water per 1 μL template or with 5 μL Promega 5× Green GoTaq® Reaction Buffer, 2.5 μL 10 mM dNTPs (Promega), 1 μL of each primer, 14.3 μL of double distilled water and 0.2 μL Promega GoTaq® G2 DNA Polymerase (Promega) per 1 μL sample (or 2 μL sample with the amount of double distilled water reduced to 13.3 μL accordingly). A Techne® Prime thermal cycler (Cole‐Parmer) or a Biometra TGradient 96 Thermocycler (Analytik Jena AG) were used. As mentioned above, all GW strains (gwPFO‐D1, gwSAT‐D1, gwBYG‐D1, gwSAC‐D1 and gwSLO‐GB1) were infected with *Wolbachia* (König, Zundel, et al., 2019), which can also be accidentally amplified with the primer pair LCO1490/HCO2198 (Bleidorn & Henze, 2021; Magnacca & Brown, 2012). Therefore, we used tetracycline‐treated endosymbiont‐free wasps for the analysis. To confirm the absence of *Wolbachia*, these strains were amplified with the *Wolbachia*‐specific primer pair W‐Specf (5‐CAT ACC TAT TCG AAG GGA TAG‐3′)/W‐Specr (5‐AGC TTC GAG TGA AAC CAA TTC‐3′) (Werren & Windsor, 2000) followed by transferring 5–10 μL of each PCR product to 1%–2% agarose gels stained by 5 μL peqgreen (VWR International GmbH) or 5 μL ROTI®‐GelStain (Carl Roth GmbH + Co. KG) and conducting gel electrophoresis. Samples were sent to Microsynth Seqlab for Sanger dideoxy sequencing.

### 2.4 Phylogenetic data analyses

Raw sequencing data were assembled, trimmed and checked for indels using GENtle v. 1.9.4. (© by Magnus Manske, University of Cologne, released under GPL 2003) (Manske, 2006). All sequences were compared to nucleotide sequences in GenBank (Benson et al., 2017) using BLAST (Altschul et al., 1990) to confirm that the correct organism had been amplified. Ambiguous positions were named according to IUPAC nomenclature. Subsequently, all sequences were aligned on nucleotide level using the multiple sequence alignment (MSA) program MAFFT v.7 (Katoh et al., 2019) with the L‐INS‐i algorithm. All sequences were transcribed into amino acids using Virtual Ribosome v. 2.0 (Wernersson, 2006) with translation table 1 (standard genetic code) for the nuclear genes and translation table 5 (invertebrate mitochondrial) for COI in order to control for unexpected stop codons or gaps. In addition to the newly collected sequence data, we further added *L. distinguendus* sequence data obtained in previous studies (König, Krimmer, et al., 2015; König, Paschke, et al., 2019) published in GenBank (Benson et al., 2017). We further added corresponding sequence data, retrieved from GenBank (Benson et al., 2017), of the outgroup species *Nasonia vitripennis* Wlk., a member of the same family as *L. distinguendus*, for COI and the nuclear genes, and *Eupelmus confusus* Al Khatib, which belongs to the same superfamily, for COI, but not the nuclear genes due to a lack of published sequences (see Appendix S1: Table A2 for overall characters and character types for all genes and Appendix S1: Table A4 for Accession Numbers of all previously published sequences).

We merged all nuclear sequences per individual into one concatenated matrix with MEGA v. X (Kumar et al., 2018). Partition homogeneity was tested and confirmed (*p* = .8) using PAUP* version 4.0a (build 169) (Swofford, 2003), and the nuclear genes were subsequently analysed as matrix, whereas the barcode segment was analysed separately. The appropriate partitioning schemes and nucleotide substitution models (see Appendix S1: Table A3) were determined with ModelFinder (Kalyaanamoorthy et al., 2017) implemented in IQ‐TREE v.1.6.12 (Nguyen et al., 2015), testing for available nuclear models. The edge‐proportional partition model (−spp, Chernomor et al., 2016) was chosen to allow for partitions evolving at different velocities and the best models for each partition were determined by the Bayesian information criterion (Schwarz, 1978). Standard settings were used for all other parameters. We inferred phylogenetic trees using the maximum likelihood optimality criterion as implemented in IQ‐TREE v.1.6.12 (Nguyen et al., 2015) with 1000 ultrafast bootstrap replicates (Hoang et al., 2018) and standard parameters. The best tree was determined by the best log‐likelihood value. FigTree v1.4.0 (http://tree.bio.ed.ac.uk/software/figtree/) was used to display the resulting phylogenetic trees along with the bootstrap support values and root them using the outgroup species. Uncorrected intra‐ and interspecific pairwise distances between the strains used for the crossing experiments were calculated using MEGA v. X (Kumar et al., 2018) with the pre‐set parameters.

### 2.5 Crossing experiments

The strain pairs BIR × OST, BIR × STU, CAN × STU, BIR × SAT and CAN × PFO were chosen to conduct the crossing experiments. These strains are representative of the DB species, the GW species and the sub‐Clades within the DB species and cover a gradient of genetic divergence (König, Paschke, et al., 2019). For each pair of strains, different reproductive barriers were studied in the four possible combinations, that is, two interstrain crossings, with females of one strain paired with males of the other strain and vice versa, and two intrastrain crossings, with females paired with males of their own strain, as controls. The intrastrain crossings were continuously conducted in parallel to the interstrain crossings at each stage to serve as control. As females of different strains have been shown to naturally differ in their acceptance of males as well as their fecundity (König, Krimmer, et al., 2015; König, Zundel, et al., 2019), data from the interstrain crosses were compared with data from intrastrain crosses with females of the same strain as controls. For crossing experiments between the strains STU and BIR, tetracycline‐treated lines were used to investigate reproductive barriers not affected by endosymbionts.

To study sexual isolation, a minimum of 20 pairs were observed for 20 min or until mating had occurred. Afterwards, both wasps were transferred onto host‐infested grains or pellets, regardless of whether copulation had occurred or not. The offered host species depended on the females in the crosses. If females originated from a GW strain, 10 g of wheat grains infested with granary weevils were provided. Females of a DB strain were offered either 10 g of wheat grains containing one drugstore beetle larva each or 5 g of koi pellets with multiple drugstore beetle larvae in each pellet. F1 offspring were counted after 4–5 weeks to assess the viability of hybrid offspring. To study sexual isolation of hybrid females, mating experiments were performed with pairs of virgin F1 females and parental‐type males as described above. To study physiological sterility and reduced fertility of hybrid females, F1 females were allowed to hatch together with F1 males to enable mating and transferred to new batches of their respective hosts, as described in detail above, for oviposition. The occurrence and numbers of total F2 offspring served as parameters for the investigated barriers. Because males in *L. distinguendus* are haploid, hybrid males do not occur until the F2 generation. To study viability of hybrid males, F1 wasps were isolated prior to hatching as described above to prevent mating and transferred to new hosts for oviposition where they only produced male offspring. The viability of hybrid males was then assessed by comparing the offspring numbers of mated F1 females producing mainly female offspring, and virgin F1 females producing only male offspring. To study behavioural and physiological sterility as well as fecundity of hybrid males, they were backcrossed to parental‐type females of both strains. Mating success served as parameter for behavioural sterility, and the occurrence and number of F3 female offspring of these crosses for sterility and reduced fertility respectively.

### 2.6 Calculation of strength of reproductive isolation

The strength of the reproductive isolation (RI) was determined based on Sobel and Chen (2014):

$$\mathrm{RI}=1-2*\frac{\left(H\right)}{\left(H\right)+\left(C\right)}$$

*H* refers to data resulting from interstrain crossings and *C* to data from intrastrain crossings. The absolute contribution (AC) of a barrier to the reproductive isolation according to their position (*n*) within the sequence of all barriers considering existing restrictions of gene flow by barriers occurring earlier in the sequence was calculated following Ramsey et al. (2003):

$$\;{\mathrm{AC}}_{n}={\mathrm{RI}}_{n}\left(1-\sum_{i=1}^{n-1}{\mathrm{AC}}_{i}\right)$$

The total isolation *T* was calculated as the sum of the absolute contributions of all barriers *m*:

$$T=\sum_{i=1}^{m}{\mathrm{AC}}_{i}$$

### 2.7 Crossing experiments testing cytoplasmic incompatibility

To test the effect of endosymbionts on hybridization, we studied crossings of the strains BIR and STU. STU has been shown to carry CI‐inducing *Spiroplasma* (Pollmann et al., 2022), whereas BIR is uninfected. Untreated individuals of the strains BIR and STU were crossed and offspring numbers in the F1 generation were analysed.

### 2.8 Sperm counts in hybrid males of CAN and STU

In addition to sexual isolation, a reduced fecundity in hybrid males was detected as very early barrier between the strains CAN and STU (see Figure 10). To study the underlying reason for this barrier, virgin males and females of these strains were crossed in all possible combinations as described above, resulting in two hybrid (CAN females × STU males, STU females × CAN males) and two control (CAN females × CAN males, STU females × STU males) crosses (*n* = 25 per combination, *N* = 100 in total). The resulting virgin F1 females were transferred to hosts to lay unfertilized male eggs. The resulting males were used for dissection of seminal vesicles or were backcrossed 2–3 days after emergence to 1‐day‐old parental‐type females according to the male strain of the original combination. After mating, females were kept isolated for 1 day to allow the sperm to move to the spermathecae. The seminal vesicles of the unmated F2 males and the spermathecae of the females from the backcrosses were dissected under a stereomicroscope (Stemi 2000; Carl Zeiss AG) using fine needles and forceps in order to examine the amount of sperm produced and transferred to females during copulation following the procedure described by Clark et al. (2010). Unmated F2 males (*n* = 100 per combination; *N* = 400 in total) were placed in a drop of Beadle–Ephrussi Ringer's solution (7.5 mg NaCl, 0.35 mg KCl, 0.27 mg CaCl_2_ per mL double distilled water), decapitated, and their aedeagi were removed along with the reproductive tissues, that is, testes, seminal vesicles and male accessory glands, which were then transferred to 20 μL of double distilled water in a depression well on a microscope slide. One seminal vesicle per male was opened and the mixture of water and released sperm cells was transferred to a 0.2 mL Eppendorf tube to be vortexed for 30 s to facilitate sperm isolation. Afterwards, eight spots of this mix were applied onto a new microscope slide and left to air‐dry for 24 h after which they were washed with 95% denatured ethanol for fixation and again left to air‐dry. To obtain sperm from spermathecae, females from the backcrosses (at least 30 per combination) were decapitated in a drop of Ringer's solution. The contents were removed from the abdomen and the spermathecae were transferred to 20 μL of double distilled water on a microscope slide with a depression well. After being isolated from the opened spermathecae, sperm was further processed as described above.

Three of the eight spots per slide were selected at random and the sperm were counted under a microscope (Axioskop 40; Carl Zeiss AG) at 100× magnification. Overall calculation was conducted by multiplying the sum of sperm cells obtained from counting the selected spots by 20 for the total volume of the water of 20 μL and dividing the result by three to control for the three spots counted. Values obtained from seminal vesicles were doubled to obtain the number of sperm for one male.

### 2.9 Statistical analyses

All statistical analyses were conducted using R v. 4.0.3 in RStudio v. 2022.07.1 (R Core Team, 2019; RStudio Team, 2016) with the pre‐installed packages as well as the packages multcomp (Hothorn et al., 2008) and car (Fox et al., 2019). Significance was assumed at *p* < .05. Binomial data, that is, occurrence data of copulation and female offspring in several generations, were analysed using a Pearson's Chi‐squared test (Pearson, 1900) for comparisons among groups and for single comparisons following a significant result if the frequencies of all observations were greater than five. Sets of binomial data with the frequency of at least one observation below five were analysed using a 2 × 4 and 2 × 6 Fisher's exact test for Count Data (Taub, 1979), respectively, for group comparisons and 2 × 2 Fisher tests for single comparisons. Single comparisons within a group were followed by Bonferroni corrections (Miller, 1981). Numerical data were analysed with linear models when data were normally distributed and variances were homogenous. If this was not the case, we used generalized linear models with the family best representing the data. All models were followed by Tukey tests (Tukey, 1949) for single comparisons. Comparisons of sperm counts between hybrid and non‐hybrid males of crossings between CAN and STU were conducted using Wilcoxon rank sum tests (Wilcoxon, 1945) with continuity correction for non‐normally distributed data and Welch two‐sample *t*‐tests (Welch, 1947) if normal distribution applied.

## 3 RESULTS

### 3.1 Phylogenetic tree reconstruction

**FIGURE 1:**
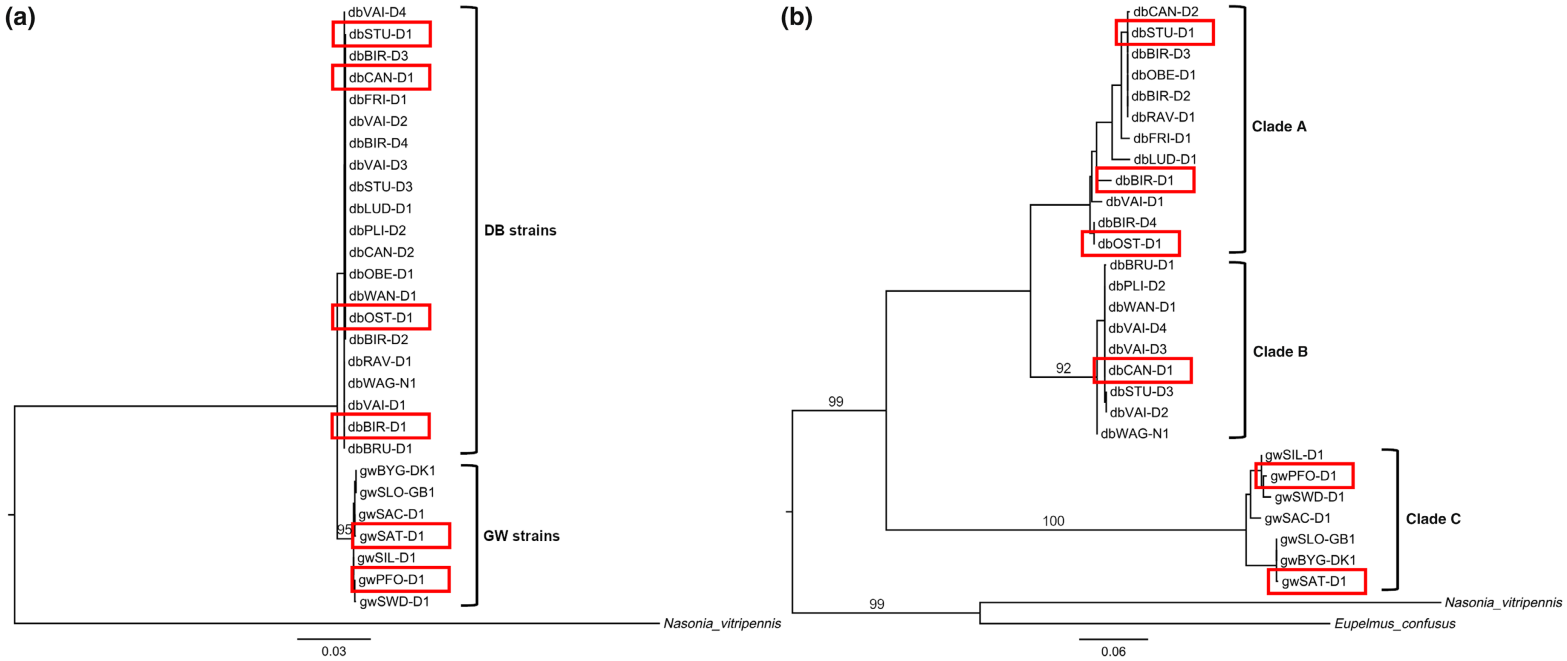
Phylogenetic trees of 28 *L. distinguendus* strains. (a) Maximum likelihood phylogenetic tree inferred from the concatenated dataset comprising the five nuclear genes CAD, ITS2 and LOC 1–3. Numbers along branches represent ML bootstrap values; values below 90 have been omitted. Brackets indicate the division of strains into two Clades representing strains collected on drugstore beetles (DB strains) and granary weevils (GW strains), res as hosts respectively. Strains in red rectangles are strains studied in crossing experiments. (b) Maximum likelihood phylogenetic tree inferred from sequences of the COI gene. Numbers along branches represent ML bootstrap values; values below 90 and within clusters with low divergence have been omitted. Brackets indicate the division of the strains into the Clades A, B and C. Strains in red rectangles are strains studied in crossing experiments.

Our phylogenetic analyses are based on five concatenated nuclear genes and the barcode segment of COI covering 28 *L. distinguendus* strains (see Appendix S1: Table A2 for overall characters and character types for all genes). The sequences of the five nuclear genes clustered into two well‐supported distinct Clades (average uncorrected pairwise distance between the Clades: 0.0101), containing all strains collected on granary weevils and drugstore beetles respectively (Figure 1a). The inferred phylogenetic tree based on the COI gene shows three well‐supported Clades which we further refer to as Clades A, B and C (Figure 1b). All strains with drugstore beetles as main hosts are divided into the two Clades A and B with an average pairwise distance of 7.17% (Figure 1b, Appendix S1: Table A6). All strains from granary weevils form Clade C which differs from the drugstore beetle Clades by 13.28% (Clade A) and 14.22% (Clade B) respectively (Appendix S1: Table A6).

### 3.2 Reproductive barriers

In the crossing experiments, we studied reproductive barriers within and between the Clades with the strains dbBIR‐D1 (‘BIR’), dbOST‐D1 (‘OST’) and dbSTU‐D1 (‘STU’) as representatives of Clade A, dbCAN‐D1 (‘CAN’) as representative of Clade B and gwSAT‐D1 (‘SAT’) and gwPFO‐D1 (‘PFO’) as representatives of Clade C.

### 3.3 Sexual isolation

**FIGURE 2:**
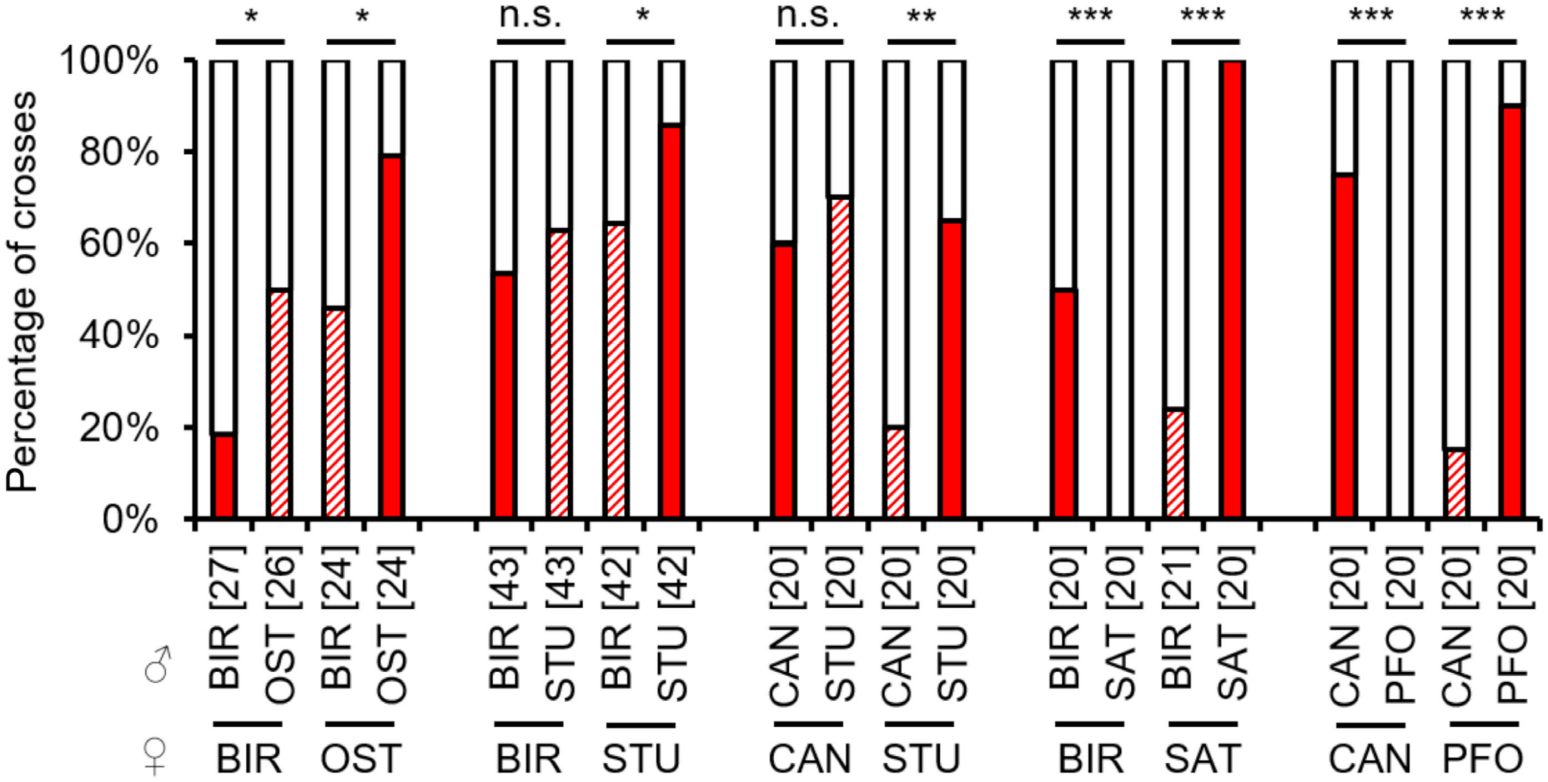
Occurrence of copulations [%] in intrastrain and interstrain crosses between females (lower strain designation) and males (upper strain designation) of several *L. distinguendus* strains. Coloured parts of the bars: the presence of copulation, filled: intrastrain, hatched: interstrain; white parts of the bars: the absence of copulation. Numbers of replicates are given in parentheses above each crossing combination. n.s. *p* > .05, *p < .05, **p < .01, ***p < .001, 2 × 2 Fisher's exact tests for Count Data (for BIR × OST, CAN × STU, BIR × SAT, and CAN × PFO) and Pearson's Chi‐squared test (for BIR × STU) for single comparisons within a group (intrastrain vs. interstrain combination) (see Tables A7–A11 for full test statistics).

In the hybrid combinations OST females × BIR males, STU females × BIR males and STU females × CAN males, the occurrence of copulations was significantly decreased compared to the respective control combinations, indicating slight sexual isolation (see Appendix S1: Tables A7–A9 for test statistics; Figure 2). In the reverse hybrid combinations, there was an increase in copulations in OST females × BIR males, and no significant differences to the controls in BIR females × STU males, and CAN females × STU males (see Appendix S1: Tables A7–A9 for test statistics; Figure 2). In the interstrain combinations BIR × SAT and CAN × PFO, there were almost no copulations (see Appendix S1: Tables A10 and A11 for test statistics; Figure 2). Therefore, subsequent barriers in the latter combination could only be investigated for CAN males and PFO females.

### 3.4 F1 female inviability

**FIGURE 3:**
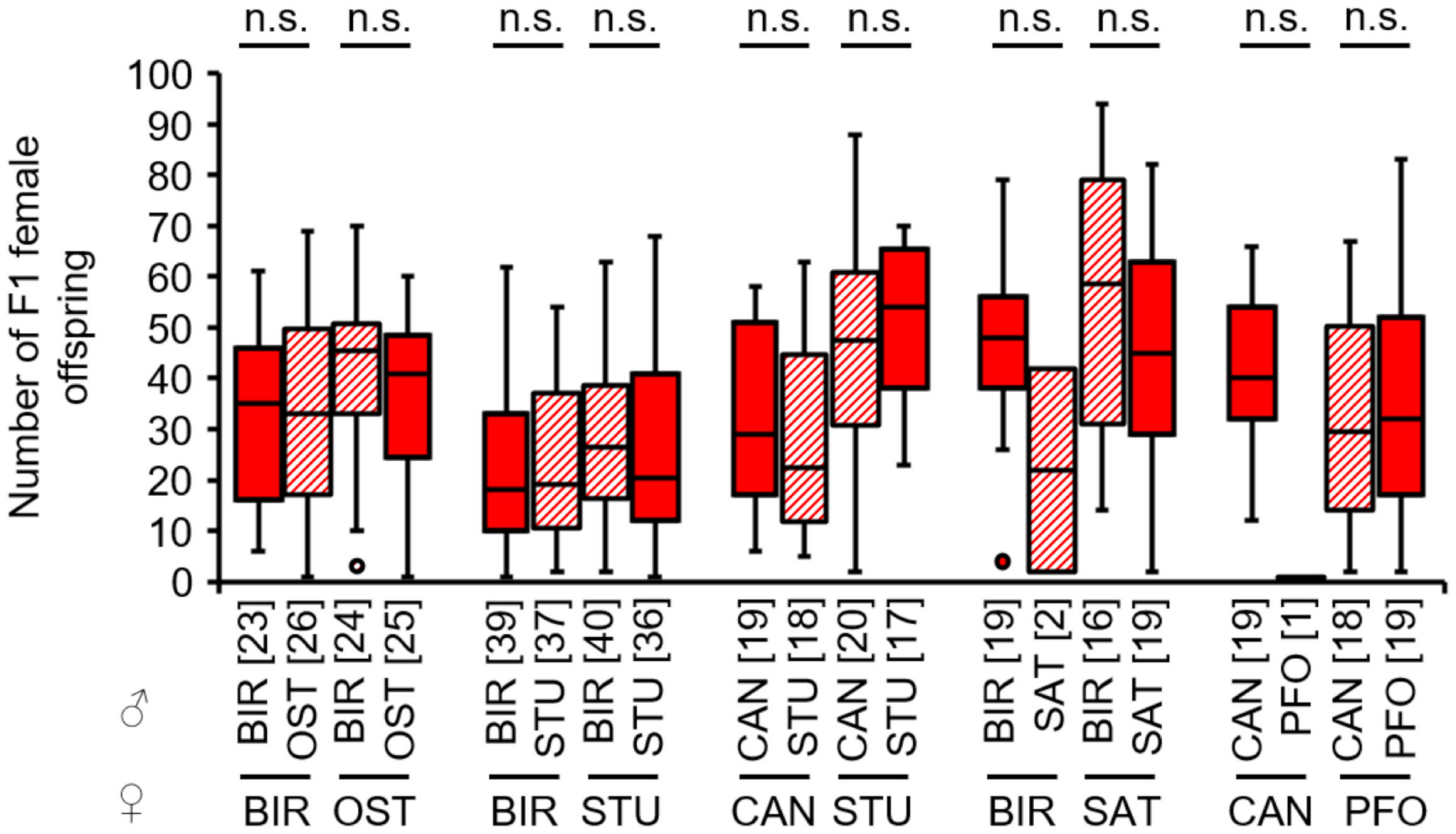
Numbers of female F1 offspring of intrastrain (filled boxes) and interstrain (hatched boxes) crosses of females (lower strain designation) and males (upper strain designation) of several *L. distinguendus* strains. Numbers of replicates are given in parentheses above each crossing combination. Only crosses with female offspring were considered. n.s. *p* > .05, BIR × OST: linear model, BIR × STU: GLM, family = quasipoisson, CAN × STU: linear model, BIR × SAT: GLM, family = quasipoisson, CAN × PFO: linear model, all models followed by Tukey tests for multiple comparisons (see Tables A12–A16 for full test statistics).

There were no significant differences in the number of F1 female offspring between intrastrain and interstrain crosses within each of the strain combinations (see Appendix S1: Tables A12–A16 for test statistics; Figure 3). Thus, non‐hybrid and hybrid F1 females are equally viable. In the crosses BIR females × SAT males and CAN females × PFO males, only a very small number of female wasps was available for testing. Most likely, this prevented significant results in these crosses.

### 3.5 F1 female behavioural sterility

**FIGURE 4:**
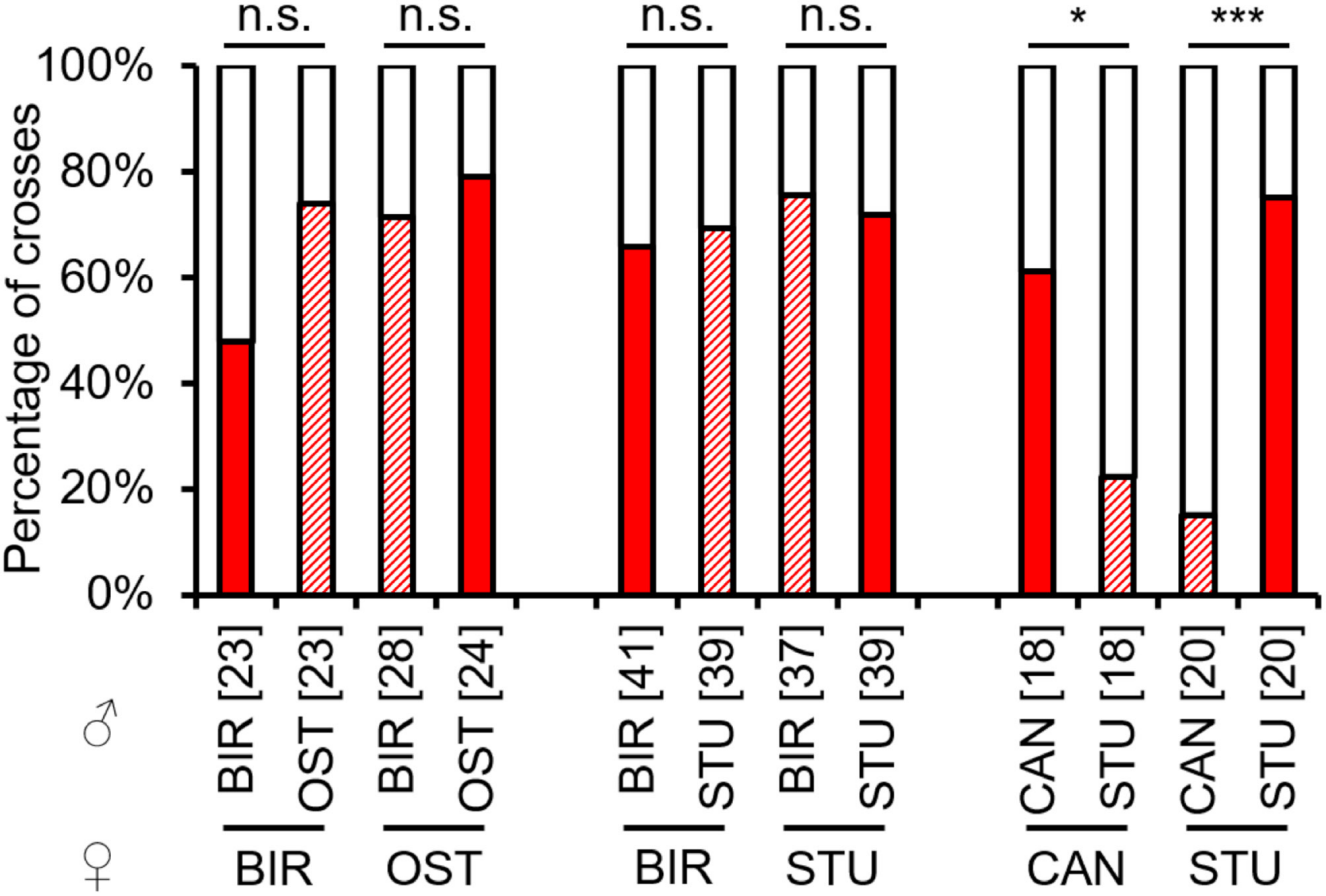
Occurrence of copulations [%] between non‐hybrid and hybrid F1 females and parental‐type males from combinations of several *L. distinguendus* strains. Strain designations on the *x*‐axis refer to the females (lower strain designation) and males (upper strain designation) of the parental cross from which the F1 females originated. Coloured parts of the bars: the presence of copulation, filled: intrastrain, non‐hybrid, hatched: interstrain, hybrid; white parts of the bars: the absence of copulation. Numbers of replicates are given in parentheses above each crossing combination. n.s. *p* > .05, *p < .05, ***p < .001, 2 × 2 Fisher's exact tests for Count Data (for BIR × OST and CAN × STU) and Pearson's Chi‐squared tests (for BIR × STU) for single comparisons within a group (non‐hybrid vs. hybrid combination) (see Tables A17–A19 for full test statistics).

Sexual isolation of F1 hybrid females was only studied for the crosses of BIR × OST, BIR × STU and CAN × STU (Figure 4). In the combinations BIR × OST and BIR × STU, no differences in the occurrence of copulations between F1 females and parental‐type males were observed (see Appendix S1: Tables A17 and A18 for test statistics). In contrast, hybrid F1 females from the combination CAN x STU had a reduced number of copulations with non‐hybrid males from the parental strains, demonstrating sexual isolation of these females (see Appendix S1: Table A19 for test statistics; Figure 4).

### 3.6 F1 female physiological sterility

**FIGURE 5:**
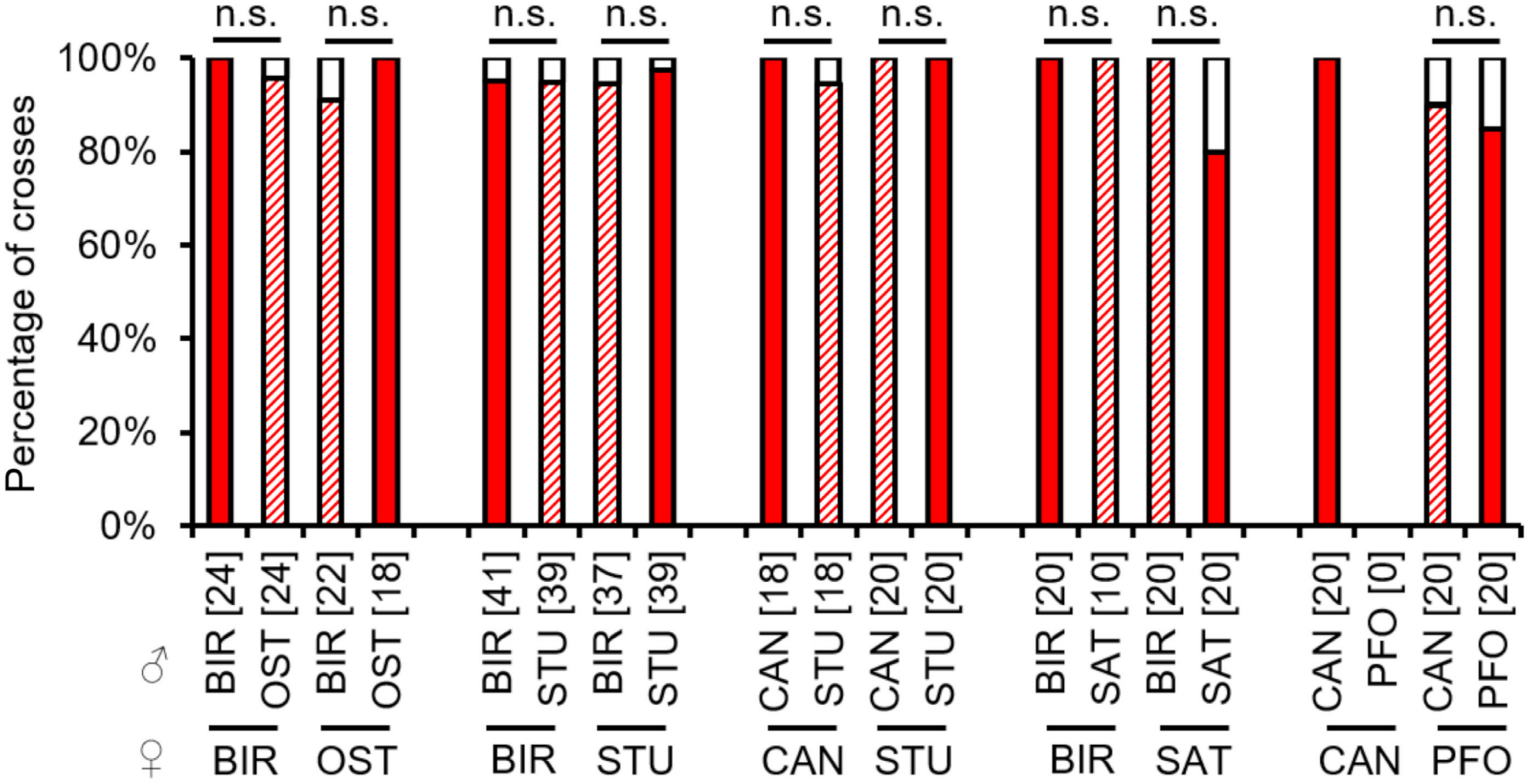
Occurrence of total F2 offspring [%] of non‐hybrid and hybrid F1 females when crossed to parental‐type males of several *L. distinguendus* strains. Strain designations on the *x*‐axis refer to the females (lower strain designation) and males (upper strain designation) of the parental cross from which the F1 females originated. Coloured parts of the bars: the presence of offspring, filled: intrastrain, non‐hybrid, hatched: interstrain, hybrid; white parts of the bars: the absence of offspring. Numbers of replicates are given in parentheses above each crossing combination. n.s. *p* > .05, 2 × 2 Fisher's exact tests for Count Data for single comparisons within a group (non‐hybrid vs. hybrid combination) (see Tables A20–A24 for full test statistics).

The occurrence of F2 offspring of F1 females mated to parental‐type males did not differ between hybrids and non‐hybrids, indicating the absence of physiological sterility in F1 hybrid females (see Appendix S1: Tables A20–A24 for test statistics; Figure 5).

### 3.7 F1 female physiological reduced fertility

**FIGURE 6:**
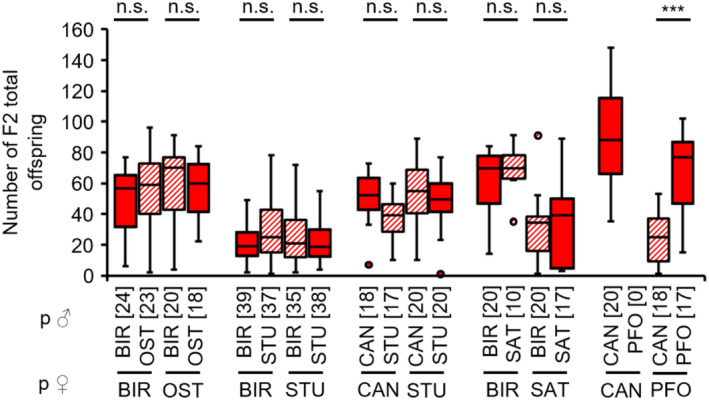
Total F2 offspring numbers of non‐hybrid (filled boxes) and hybrid (hatched boxes) F1 females when crossed to parental‐type males of several *L. distinguendus* strains. Strain designations on the *x*‐axis refer to the females (lower strain designation) and males (upper strain designation) of the parental cross from which the F1 females originated. Numbers of replicates are given in parentheses above each crossing combination. Only crosses with offspring were considered. n.s. *p* > .05, ***p < .001, BIR × OST: GLM, family = quasipoisson, BIR × STU: GLM, family = quasipoisson, CAN × STU: linear model, BIR × SAT: GLM, family = quasipoisson, CAN × PFO: linear model, all models followed by Tukey tests for multiple comparisons (see Tables A25–A29 for full test statistics).

There were no significant differences in the number of total F2 offspring from non‐hybrid and hybrid F1 females of the combinations BIR × OST, BIR × STU, CAN × STU and BIR × SAT (see Appendix S1: Tables A25–A28 for test statistics; Figure 6). In contrast, hybrid F1 females of the combination PFO females × CAN males had significantly reduced F2 total offspring numbers compared to non‐hybrid control females (see Appendix S1: Table A29 for full test statistics; Figure 6). No hybrid F1 females of the combination CAN females × PFO males were available.

### 3.8 F2 male inviability

**FIGURE 7:**
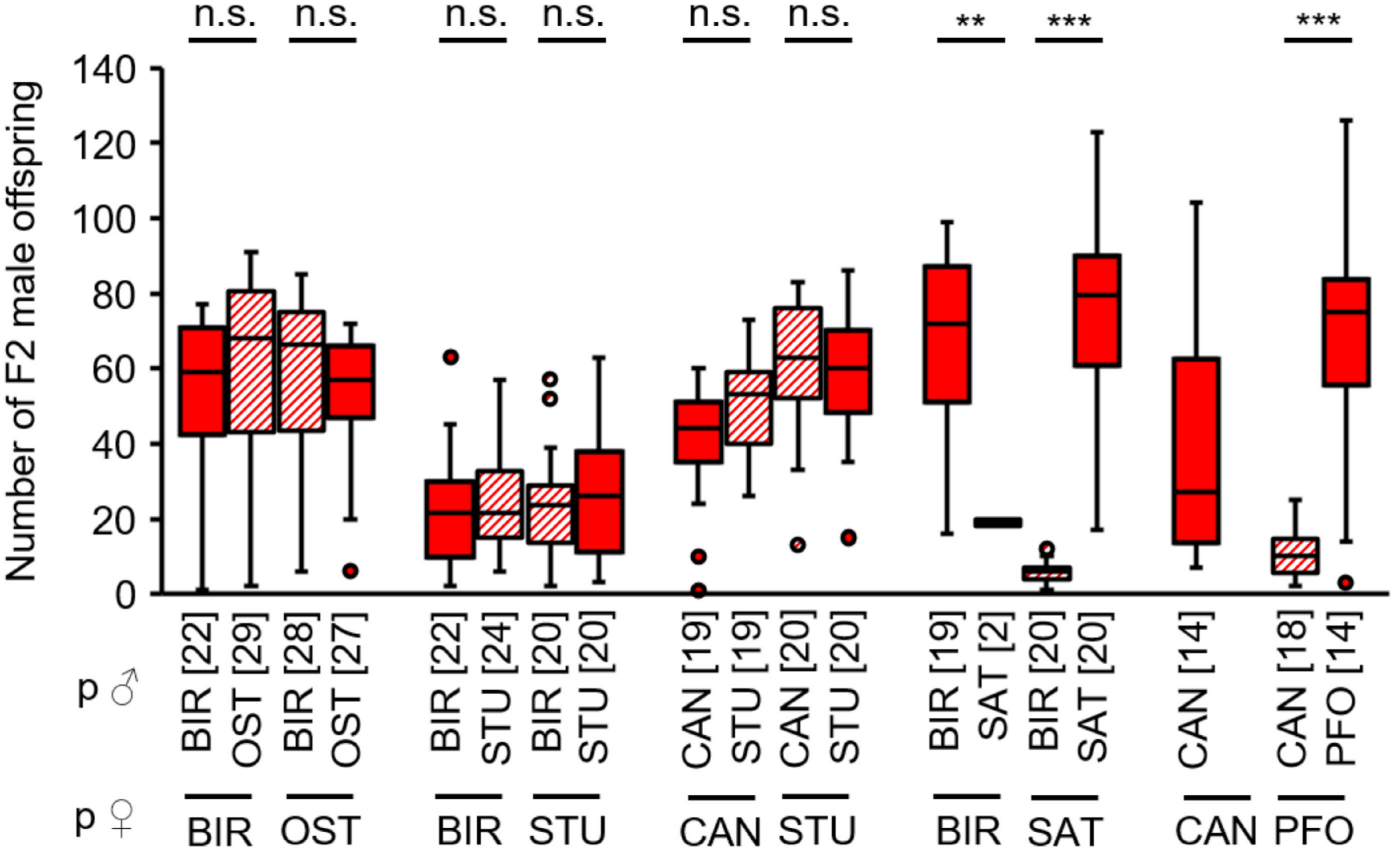
F2 male offspring numbers of virgin non‐hybrid (filled boxes) and hybrid (hatched boxes) F1 females of several *L. distinguendus* strains. Strain designations on the *x*‐axis refer to the females (lower strain designation) and males (upper strain designation) of the parental cross from which the F1 females originated. Numbers of replicates are given in parentheses above each crossing combination. Only crosses in which females produced offspring were considered. n.s. *p* > .05, **p < .01, *** *p* < .001, BIR × OST: GLM, family = quasipoisson, BIR × STU: GLM, family = negative binomial, CAN × STU: GLM, family = quasipoisson, BIR × SAT: linear model, CAN × PFO: GLM, family = quasipoisson, all models followed by Tukey tests for multiple comparisons (see Tables A30–A34 for full test statistics).

F2 male inviability was studied with the offspring of virgin non‐hybrid and hybrid females, which produce only males. Combinations BIR × OST, BIR × STU and CAN × STU did not show any significant differences in numbers of F2 male offspring between non‐hybrid and hybrid F1 females and therefore no hybrid male inviability (see Appendix S1: Tables A30–A32 for test statistics; Figure 7). In contrast, for the combination BIR × SAT, the numbers of hybrid F2 male offspring were significantly reduced compared to the control crosses, indicating hybrid male inviability (see Appendix S1: Table A33 for test statistics; Figure 7). Likewise, there were less hybrid F2 male offspring from hybrid PFO × CAN females (see Appendix S1: Table A34 for test statistics; Figure 7).

### 3.9 F2 male behavioural sterility

**FIGURE 8:**
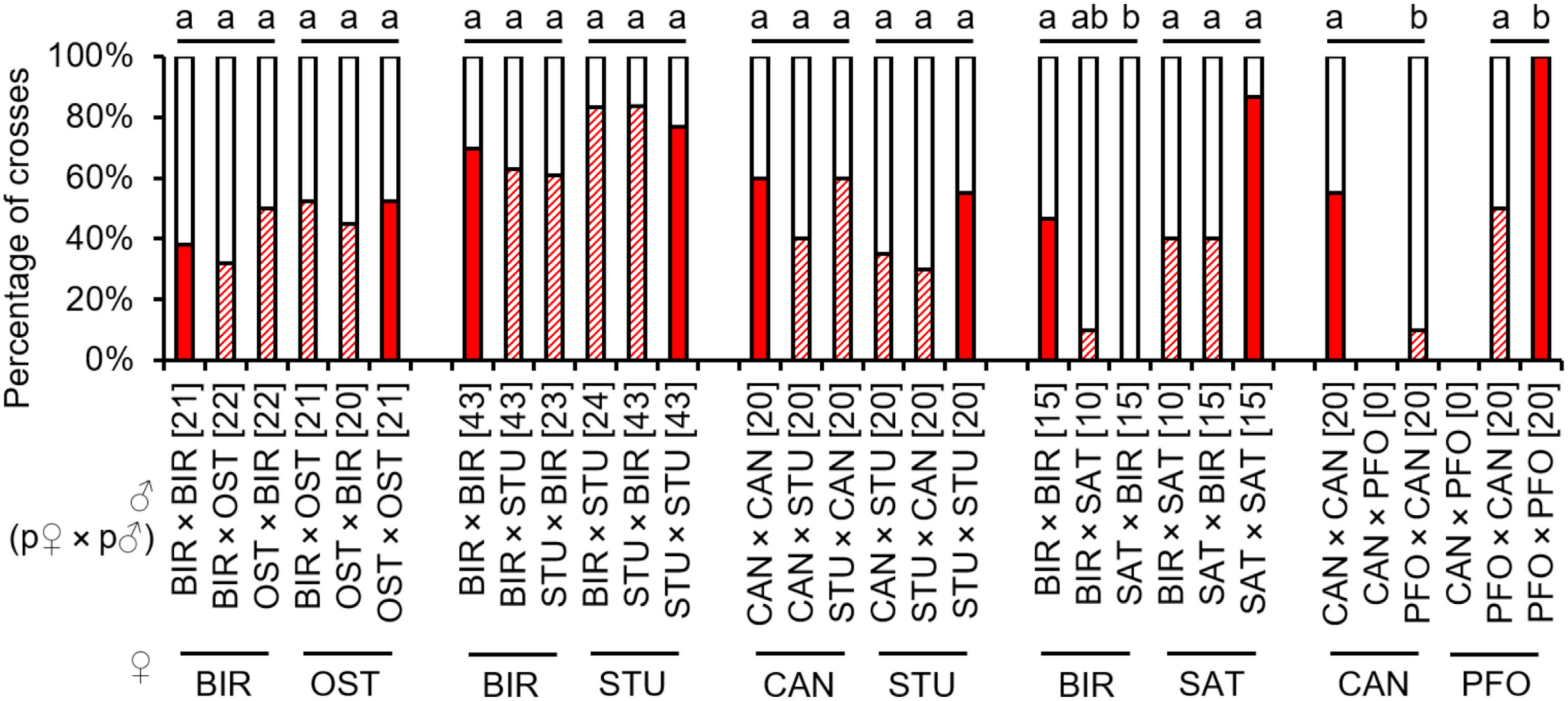
Occurrence of copulations [%] in backcrosses of F2 non‐hybrid and hybrid males to parental‐type females of several *L. distinguendus* strains. Strain designations on the *x*‐axis refer to the parental‐type females (lower strain designation) and the parental cross from which the F2 males originated (upper strain designation). Coloured parts of the bars: the presence of copulation, filled: non‐hybrid, hatched: hybrid; white parts of the bars: the absence of copulation. Numbers of replicates are given in parentheses above each crossing combination. Different lowercase letters indicate statistical differences, 2 × 3 Fisher's exact tests for Count Data (for BIR × STU and BIR × SAT), Pearson's Chi‐squared tests (for BIR × OST and CAN × STU), and 2 × 2 Fisher's exact tests for Count Data (for CAN × PFO) for group comparisons (non‐hybrid vs. hybrid males crossed to parental‐type females of the same strain), 2 × 2 Fisher's exact tests for Count Data followed by Bonferroni correction for single comparisons after significant differences in group comparisons within a strain combination (see Tables A35–A39 for full test statistics).

The occurrence of copulations in backcrosses with parental‐type females did not differ significantly between hybrid and non‐hybrid F2 males from all strain combinations of BIR × OST, BIR × STU and CAN × STU, demonstrating the absence of behavioural sterility. Likewise, there were no differences between hybrid SAT × BIR males (both combinations) crossed to SAT females, as well as hybrids of BIR females × SAT males crossed to BIR females (see Appendix S1: Tables A35–A37 for test statistics; Figure 8). Hybrid F2 males of SAT females × BIR males crossed to BIR females and the available crosses from PFO × CAN had much fewer copulations compared to the control males when backcrossed to both parental females (see Appendix S1: Tables A38 and A39 for test statistics; Figure 8).

### 3.10 F2 male physiological sterility

**FIGURE 9:**
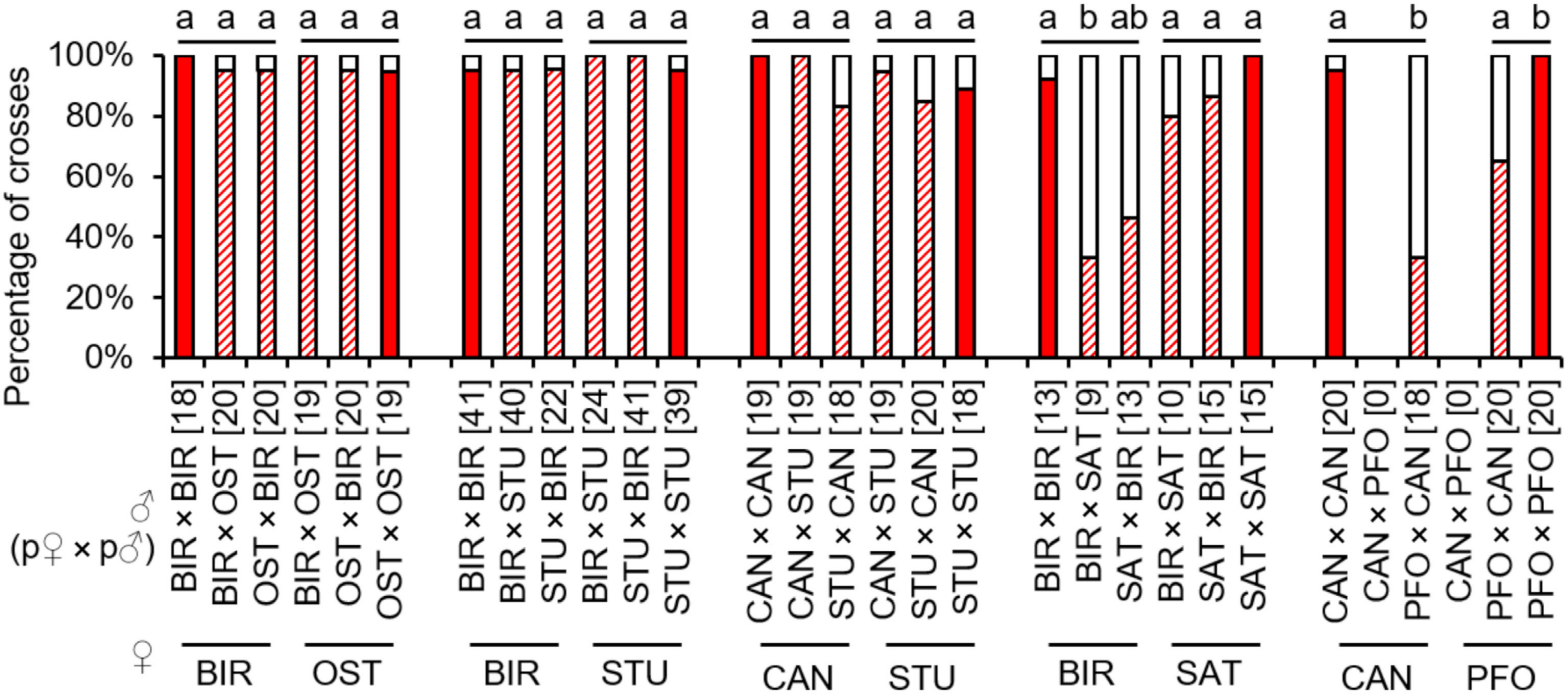
Occurrence of female F3 offspring [%] in backcrosses of non‐hybrid and hybrid F2 males to parental‐type females of several *L. distinguendus* strains. Strain designations on the *x*‐axis refer to the parental‐type females (lower strain designation) and the parental cross from which the F2 males originated (upper strain designation). Coloured parts of the bars: the presence of female offspring, filled: non‐hybrid, hatched: hybrid; white parts of the bars: the absence of female offspring. Numbers of replicates are given in parentheses above each crossing combination. Different lowercase letters indicate statistical differences, 2 × 3 Fisher's exact tests for Count Data (for BIR × OST, BIR × STU, CAN × STU and BIR × SAT) and 2 × 2 Fisher's exact tests for Count Data (for CAN × PFO) for group comparisons (non‐hybrid vs. hybrid males crossed to parental‐type females of the same strain), 2 × 2 Fisher's exact tests for Count Data followed by Bonferroni correction for single comparisons after significant differences in group comparisons within a strain combination (see Tables A40–A44 for full test statistics).

The percentage of crosses with female F3 offspring did not differ significantly in backcrosses of hybrid and non‐hybrid F2 males to parental‐type females for the strain combinations BIR × OST, BIR × STU, CAN × STU and BIR × SAT. Obviously, these hybrid males are able to produce female offspring and are not physiologically sterile (see Appendix S1: Tables A40–A42 for test statistics; Figure 9). However, the percentage of crosses with female offspring was significantly reduced as compared to controls in hybrid F2 males from the combination BIR females × SAT males crossed to BIR females, and of the parental cross PFO females × CAN males. Some of these hybrids are physiologically sterile (see Appendix S1: Tables A43 and A44 for test statistics; Figure 9).

### 3.11 F2 male reduced fertility or F3 female inviability

**FIGURE 10:**
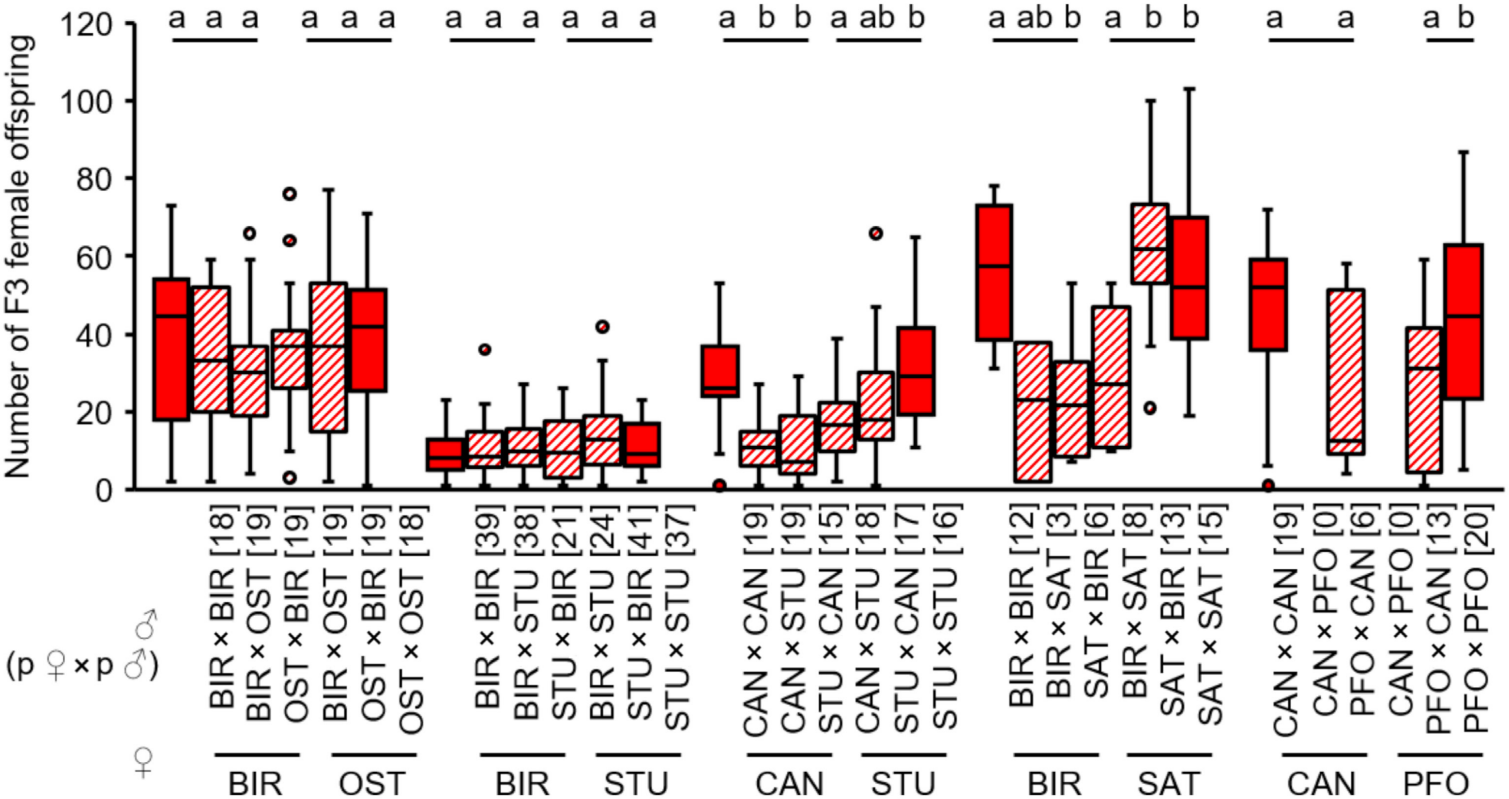
F3 female offspring numbers of non‐hybrid (filled boxes) and hybrid (hatched boxes) F2 males when backcrossed to parental‐type females of several *L. distinguendus* strains. Strain designations on the *x*‐axis refer to the parental‐type females (lower strain designation) and the parental cross from which the F2 males originated (upper strain designation). Numbers of replicates are given in parentheses above each crossing combination. Only crosses with female offspring were considered. Different lowercase letters indicate statistical differences, BIR × OST: linear model, BIR × STU: GLM, family = negative binomial, CAN × STU: GLM, family = quasipoisson, BIR × SAT: linear model, CAN × PFO: linear model, all models followed by Tukey tests for multiple comparisons (see Tables A45–A49 for full test statistics).

A reduced number of F3 female offspring numbers compared to controls was observed in hybrid F2 males when backcrossed to parental‐type females in all strain combinations of BIR × OST and BIR × STU (see Appendix S1: Tables A45 and A46 for test statistics; Figure 10). In contrast, hybrid F2 males from several combinations of STU × CAN, BIR × SAT and CAN × PFO sired significantly fewer F3 females than control F2 males. This likely indicates a reduced fertility of these hybrid males (see Appendix S1: Tables A47–A49 for test statistics; Figure 10). However, we cannot exclude the possibility that this result is in fact caused by an increased inviability of hybrid F3 females.

### 3.12 The role of CI due to endosymbionts in BIR × STU

To examine the role of CI as barrier between BIR and STU, the number of F1 female offspring was studied with wasps that were not treated with antibiotics, and one additional treatment with tetracycline‐fed STU males. Thereby, a reduction in the occurrence of female offspring was observed in crosses between BIR females and STU males compared to the non‐hybrid control combination and the hybrid combination with BIR females and tetracycline‐fed STU males. There was no reduction of female offspring when STU females were crossed to BIR males (see Appendix S1: Tables A50 and A51 for full test statistics; Table 2). This demonstrates the presence of CI as unilateral isolating barrier between BIR females and STU males.

**TABLE 2:** Numbers of crosses with and without female offspring and percentage of crosses with female offspring for all possible combinations with untreated individuals of the strain combination BIR × STU as well as the crossing combination BIR females × tetracycline‐treated (tet) STU males.

Crossing combination	*n*	Crosses with female offspring	Crosses without female offspring	% Crosses with female offspring	Statistical difference
BIR ♀ × BIR ♂	21	21	0	100	a
BIR ♀ × STU_tet_ ♂	39	39	0	100	a
BIR ♀ × STU ♂	40	19	21	47.5	b
STU ♀ × BIR ♂	38	33	5	86.8	a
STU ♀ × STU ♂^a^	33	29	4	87.9	a

*Note*: Different lowercase letters indicate statistical difference at *p* < .05 (see Appendix S1: Tables A50 and A51 for test statistics).

^a^ Data from Pollmann et al. (2022).

### 3.13 Isolation indices

For the strain combination BIR × OST, all isolation indices except for one were close to zero, indicating random gene flow (Table 3). Only for sexual isolation as barrier, significant indices of −0.44 and 0.27 were found for the combinations BIR females × OST males, and OST females × BIR males respectively. Thus, interstrain matings occurred about 40% more often in the combination BIR females × OST males and were about 30% less likely in the combination OST females × BIR males than intrastrain matings. Similarly, the isolation indices in the strain combination BIR × STU with wasps treated with antibiotics, that is, in the absence of CI, diverged only little from zero in either direction, ranging from −0.14 to 0.14. There was only very slight, but significant sexual isolation in the combination STU females × BIR males. In contrast, with wasps which have not been treated with antibiotics, CI caused a hybrid female inviability index of 0.356. Regardless of CI, total isolation between BIR and STU was −0.28 and 0.14 respectively. Isolation indices in the combination CAN × STU were high for several barriers, that is, for sexual isolation of the parental cross STU females × CAN males, for hybrid F1 females of both combinations, and with respect to reduced fertility of hybrid males. The combined barriers between these strains resulted in a total isolation of 0.8319 and 0.9651, respectively, indicating very high to near complete isolation (Table 3). For the strain combinations BIR × SAT and CAN × PFO, isolation indices, except for reduced fertility of hybrid females, were consistently ranging between intermediate and high values. Sexual isolation in these combinations was close to complete, although the occurrence of females in the F1 generation suggests that copulation did occur in some crosses (Table 3). The resulting total isolation indices of 1.0 and 0.996 for BIR × SAT, and 1.0 and 0.9991 for CAN × PFO (Table 3) suggest the absence of gene flow between the strains.

**TABLE 3:** Strength of reproductive isolation per barrier for all crossing combinations given as indices ranging from −1 (outcrossing is favoured), over 0 (random crossing) to 1 (complete isolation), sorted by % difference in the COI barcode.

Barrier	BIR ♀ × OST ♂	OST ♀ × BIR ♂	BIR ♀ × STU ♂	STU ♀ × BIR ♂	CAN ♀ × STU ♂	STU ♀ × CAN ♂	BIR ♀ × SAT ♂	SAT ♀ × BIR ♂	CAN ♀ × PFO ♂	PFO ♀ × CAN ♂
% COI difference	1.7%		2.8%		7.2%		13.9%		14.0%	
Ecological isolation	N/A						Present		N/A	
Sexual isolation parentals	−0.44	**0.27**	−0.08	**0.14**	−0.08	**0.53**	**1.0**	**0.62**	**1.0**	**0.71**
Inviability hybrid ♀ (without CI)	0.03	−0.05	−0.03	0.13	0.13	0.06	**0.37**	−0.13	0.95^a^	0.04
Inviability hybrid ♀ (with CI)	–	–	**0.356**	0.006	–	–	–		–	
Behavioural sterility hybrid ♀	−0.19	0.08	−0.02	−0.03	**0.47**	**0.67**	Not studied		Not studied	
Physiological sterility hybrid ♀	0.02	0.04	0	0.01	0.03	0	0	−0.11	N/A	−0.03
Reduced fertility hybrid ♀	−0.02	−0.08	−0.14	−0.06	0.14	−0.05	0.0	0.06	N/A	**0.51**
Inviability hybrid ♂	−0.07	−0.08	0	0.05	−0.09	−0.02	**0.58**	**0.86**	N/A	**0.76**
Behavioural sterility hybrid ♂^b^	0.05	−0.03	0.01	0.01	0.21	0.15	**0.51**	**0.68**	N/A	**0.51**
Physiological sterility hybrid ♂	0.0	0.01	−0.01	−0.01	−0.02	0.06	**0.29**	**0.20**	N/A	**0.35**
Reduced fertility hybrid ♂	0.09	0.13	−0.01	−0.1	**0.34**	**0.40**	**0.37**	**0.23**	N/A	**0.40**
**Total isolation (without CI)**	−0.5276	0.2949	−0.2785	0.1436	**0.8319**	**0.9651**	**1.0**	**0.996**	**1.0**	**0.9991**
**Total isolation (with CI)**	–	–	0.0860	0.1498	–	–	–	–	–	–

*Note*: Indices which are based on significant differences between non‐hybrid and hybrid crosses are given in bold.

Abbreviation: N/A, not available.

^a^ *n* = 1.

^b^ From behavioural sterility of hybrid males onward: means of indices were provided, calculated from data of backcrosses to both wildtype strain females.

### 3.14 Sperm counts

To investigate if the reduced fertility of hybrid males in the combination CAN × STU is due to a reduced number of sperm cells produced by the males and/or transferred to females during copulation, sperm counts in seminal vesicles and spermathecae were compared in hybrid and non‐hybrid males and females after mating. The amount of sperm cells in the seminal vesicles of hybrid males of the parental cross STU females × CAN males was significantly lower than in those of the control cross. This was not the case in hybrid males from the parental combination CAN females × STU males (see Table A52 for full statistics; Table 4). However, sperm retrieved from spermathecae did not differ in numbers between females mated to males of either combination or controls (see Table A52 for full statistics; Table 4).

**TABLE 4:** Sperm counts in seminal vesicles of unmated F2 males obtained and in spermathecae of females mated to F2 males.

	Parental cross of F2 ♂	♀	*n*	Median	Quartile 1	Quartile 3	Statistical difference
Seminal vesicles of males	CAN ♀ × CAN ♂	–	100	3163.335	2598.3325	3724.975	*p* = .00***
	STU ♀ × CAN ♂	–	100	2363.33	1680.0025	3050	
	CAN ♀ × STU ♂	–	100	1986.67	1383.3325	2461.6675	*p* = .58n.s.
	STU ♀ × STU ♂	–	100	1800	1301.67	2546.67	
Spermathecae of females after copulation	CAN ♀ × CAN ♂	CAN	32	380	280	454.9975	*p* = .14n.s.
	STU ♀ × CAN ♂	CAN	34	300	214.9975	424.9975	
	CAN ♀ × STU ♂	STU	32	433.33	310	596.67	*p* = .40n.s.
	STU ♀ × STU ♂	STU	31	393.33	266.67	576.665	

*Note*: Pairwise statistical comparisons were conducted according to the origin strain of the males (***p < .001, n.s. *p* > .05; see Appendix S1: Table A52 for full statistics).

## 4 DISCUSSION

In our study we investigated the diversity within the species complex of *L. distinguendus* by conducting phylogenetic tree inferences with five nuclear genes and the COI gene, and studied reproductive isolation and isolation barriers according to the biological species concept (BSC) in crossing experiments to identify separate species within the complex.

### 4.1 *L. distinguendus* is split into three reproductively isolated Clades

The phylogenetic tree inference based on nuclear loci recognized two Clades, which agree with the preference for either *S. granarius* or *S. paniceum* as hosts. They also match ‘*Stegobium* Clade I’ and ‘*Sitophilus* Clade I’ from our earlier study which was based on the same nuclear genes, another part of the COI gene, and only nine of the 28 *L. distinguendus* strains presented here (König, Krimmer, et al., 2015; König, Paschke, et al., 2019). In contrast, our newly reconstructed COI phylogenetic tree displays a more distinct topology and clusters into three well‐supported Clades. Of these, two comprise strains which were collected in households and/or with drugstore beetles as baits (Clades A and B). From these, Clade A largely equals the ‘*Stegobium* Clade I’ from our previous study (König, Paschke, et al., 2019), while Clade B consists mostly of strains which were collected only recently and were unknown to us when we conducted the earlier study. The third Clade (Clade C) comprises strains with granary weevils as preferred hosts and is identical to the ‘*Sitophilus* Clade I’ (König, Krimmer, et al., 2015; König, Paschke, et al., 2019). Discordances between the resolution and topology of phylogenetic trees recovered from mitochondrial and nuclear genes are common (e.g. Gebiola et al., 2012; Hernández‐López et al., 2012) and can be caused by higher rates of evolution of mitochondrial DNA compared to nuclear DNA (Brown et al., 1979; Hubert & Hanner, 2015), for example due to incomplete lineage sorting (Gebiola et al., 2012). This difference in evolutionary rates has been shown to be especially pronounced in Hymenoptera (Kaltenpoth et al., 2012). Alternatively, a high diversity in mtDNA can result from a high diversity in endosymbionts in arthropod populations even though the diversity in nuclear genes is low (Hurst & Jiggins, 2005). This is because endosymbionts, such as *Wolbachia*, can link to specific mtDNA types, causing them to sweep through a population along with the endosymbionts (reviewed by Hurst & Jiggins, 2005). In fact, different infections with endosymbiotic bacteria have been detected within the *L. distinguendus* species complex (König, Zundel, et al., 2019; Pollmann et al., 2022, also see Table A1) that could have influenced our results. Therefore, to study whether Clade B within our COI tree is in fact separate from the other Clades, we studied reproductive isolation based on the BSC between Clades A, B and C. This confirmed the COI‐tree topology presented here. The three COI clusters are reproductively isolated groups, with almost complete reproductive isolation between Clade A (represented by strain STU) and Clade B (represented by strain CAN), ranging from 0.83 to 0.97, and complete reproductive isolation between Clade A (represented by strain BIR) and Clade C (represented by strain SAT), and between Clade B (represented by strain CAN) and Clade C (represented by strain PFO). As we consider the values of total reproductive isolation of 0.83–1.0 to be ‘substantial reproductive isolation’ (see Coyne & Orr, 2004), these Clades constitute separate species according to the BSC. While Clades A and C have been shown to be reproductively isolated before (König, Krimmer, et al., 2015; König, Zundel, et al., 2019), here, we also demonstrate that Clade B, so far considered to belong to Clade A based on a phylogeny gained from a single locus analysed in a single strain (König, Paschke, et al., 2019), is in fact a new group reproductively isolated from Clade A and Clade C. Thus, the *L. distinguendus* species complex does not only comprise two (König, Zundel, et al., 2019), but at least three distinct species. An outlier Clade, designated as ‘*Stegobium* Clade II’, which was detected in an earlier study (König, Paschke, et al., 2019), might constitute yet another separate species. However, this Clade was not incorporated into the present study as the laboratory strains had been lost beforehand. As there are no discernible morphological differences, at least between the most strongly separated Clades A and C (Wendt et al., 2014), *L. distinguendus* seems to be another example for cryptic diversity. This is common in parasitic Hymenoptera and Chalcidoidea in particular, (e.g. Darwell & Cook, 2017; Heraty et al., 2007; Hernández‐López et al., 2012; Kenyon et al., 2015; Stahlhut et al., 2013), which supports the hypothesis that Hymenoptera are the most diverse order within the animal kingdom (Forbes et al., 2018). Remarkably, all species in the *L. distinguendus* complex, including the newly discovered cryptic species, occur in close contact to human habitations.

### 4.2 Results from barcode data do not agree with data based on the BSC

The BSC (Mayr, 1969) defines species as populations which are readily interbreeding with each other, but not with other populations. Applied for species delimitation, it therefore requires the existence of complete (Mayr, 1969) or very strong (Coyne & Orr, 2004) reproductive isolation caused by sexual isolation, partial or total inviability, sterility and/or reduced fertility of hybrids (Coyne & Orr, 2004) to justify the assumption of separate species (Coyne & Orr, 2004; Mayr, 1969). Remarkably, while the BSC is the most prominent species concept in text books (Barton et al., 2007; Futuyma, 2018) and predominantly used by researchers focusing on study areas such as ecology and evolution, it is rarely used in taxonomy and phylogenetics, the scientific fields where species are described (Stankowski & Ravinet, 2021). In these fields, mostly molecular differences are used for species delimitation, sometimes combined with morphological and/or ecological data in an integrative taxonomy approach (Schlick‐Steiner et al., 2010). Thereby, species are often separated based on the divergence of the barcode segment (Hajibabaei et al., 2006; Hebert et al., 2004; Smith et al., 2005, 2006; Ward et al., 2005), that is, a gap between intraspecific variance and interspecific distance, referred to as barcode gap (Hebert et al., 2004; Meyer & Paulay, 2005). Although different methods have been suggested to set the threshold for the barcode gap (e.g. Hebert et al., 2004), a threshold of 2% difference in COI is often used to support the assumption of separate species, especially for parasitoid wasps (Fernández‐Flores et al., 2013; Smith et al., 2013; Stahlhut et al., 2013) (e.g. Fagan‐Jeffries et al., 2018; Kang et al., 2017; Meierotto et al., 2019; Sharkey et al., 2021; Smith et al., 2013, reviewed by Hubert & Hanner, 2015). Likewise, the BIN system used by the BOLD database uses a 2.2% threshold of COI difference (Ratnasingham & Hebert, 2013). This strategy was successful to recover taxonomies which were established based on other traits like morphology and ecology in different groups of organisms and to reveal cryptic diversities within the studied groups (Hajibabaei et al., 2006; Hebert et al., 2004; Smith et al., 2005, 2006; Ward et al., 2005). In extreme cases, up to hundreds of presumed new species were described largely based on barcodes, which has been termed turbo‐taxonomy (Butcher et al., 2012; Meierotto et al., 2019; Sharkey et al., 2021).

Comparing this method of species delimitation to the results of our crossing experiments with *L. distinguendus* reveals that the strictly set thresholds commonly used for species delimitation based on barcodes, specifically thresholds of 2% or 2.2% COI divergence (Fagan‐Jeffries et al., 2018; Kang et al., 2017; Meierotto et al., 2019; Ratnasingham & Hebert, 2013; Sharkey et al., 2021; Smith et al., 2013) do not match the species limits determined based on the BSC. If a COI difference of 2% or 2.2% would be accepted as sufficient to declare two populations as distinct species, the strains BIR and STU, which differ in COI by 2.8%, would have to be considered as separate species. However, as there is only little reproductive isolation between BIR and STU, this hypothesis must be rejected. In contrast, the threshold for species delimitation in *L. distinguendus* presumably lies well above the 2% and 2.2% COI difference, as the least divergent species pair with substantial reproductive isolation has a COI difference of 7.2% (CAN × STU). Therefore, our results support the criticism towards relying on universally fixed barcoding gaps for species delimitation and species description (Ahrens et al., 2021; DeSalle, 2006; Meier et al., 2022; Meyer & Paulay, 2005; Wiemers & Fiedler, 2007; Zamani et al., 2021, 2022). Because the divergence and threshold values for species delimitation are likely to be taxon specific (Gadawski et al., 2022; Hebert et al., 2003; Huang et al., 2008; Phillips et al., 2022), based on our results we propose to calibrate species delimitation thresholds based on barcoding and morphology by studying reproductive barriers according to the BSC in suitable related species. This should be particularly easy in parasitoids, many species of which are bred in laboratories as they can be used as biological control agents (Cherif et al., 2021; Gabarra et al., 2015; Ibouh et al., 2019; Martel et al., 2019; Ovruski & Schliserman, 2012; Postali Parra & Coelho, 2019; Quicke, 1997; Smith, 1996; Waage & Hassell, 1982; Wang et al., 2019), making them easily accessible for such experiments.

### 4.3 Emergence of reproductive barriers during the process of speciation in *L. distinguendus*

While the process of speciation and the order of emergence of reproductive barriers was intensively studied in recent years for a large number of taxa (Coyne & Orr, 1989; Fitzpatrick et al., 2009; Mendelson et al., 2007; Seehausen et al., 2014; Xue et al., 2009), only very few studies have dealt with speciation in Hymenoptera. Because our strains of *L. distinguendus* represent different stages within the continuum from closely related populations over incipient to distinct species, they can provide valuable insight into the emergence of reproductive barriers during speciation in parasitoid hymenopterans.

### 4.4 Sexual isolation

Our results indicate that sexual isolation, albeit weak and unilateral, emerges as first barrier between closely related strains without obvious ecological separation, such as the use of different hosts, and increases in strength during the process of separation. This agrees with other data on reproductive barriers in *L. distinguendus* (König, Zundel, et al., 2019), and with the recent study on reproductive barriers within a population of *N. vitripennis* (Malec et al., 2021), where slight sexual isolation was found between closely related populations and even within a population of the same species. As in *N. vitripennis*, sexual isolation in *L. distinguendus* is most likely caused by a mate choice decision of the female, which do not accept males with diverging mandibular pheromones that are applied on the female antennae during courtship (König, Seeger, et al., 2015; Ruther & Hammerl, 2014). The findings that sexual isolation as reproductive barrier seems to precede ecological isolation challenge the view that sexual selection is only a by‐product of natural selection and should be dropped as unique speciation mechanism (Ritchie, 2007; Rundle & Rowe, 2018; Safran et al., 2013; Scordato et al., 2014; Weissing et al., 2011). In *N. vitripennis*, we found evidence that inbreeding between sisters and brothers, which is common in Chalcidoidea and also occurs in *L. distinguendus*, might lead to sexual isolation by genetic drift (Uyeda et al., 2009) and promote speciation similar to geographic barriers in allopatric populations, as hypothesized by Askew (Askew, 1968; Malec et al., 2021). However, detailed studies on the ecology of the strains studied here, such as the thorough investigation of host choice and host use, are required to answer the question if sexual isolation precedes ecological isolation in *L. distinguendus*.

### 4.5 Cytoplasmic incompatibility (CI)

Following sexual isolation, CI, the incompatibility of sperm and egg (Yen & Barr, 1971), was identified as second barrier between the closely related strains BIR and STU. Generally, CI can occur in arthropods in crossings between uninfected females and males infected with specific endosymbionts (Barr, 1980; Breeuwer & Werren, 1990). In haplodiploids like *L. distinguendus* it appears as a reduction in the number or the complete absence of female offspring (Ryan & Saul, 1968). Endosymbionts which are able to induce CI are *Wolbachia* (Yen & Barr, 1971), *Candidatus* Cardinium hertigii (Hunter et al., 2003), a new bacterium in the same family as *Wolbachia*, called *Candidatus* Mesenet longicola (Takano et al., 2017, 2021), and a strain of the Gammaproteobacterium *Rickettsiella* (Rosenwald et al., 2020). For *L. distinguendus*, we have recently shown that the STU strain carries the endosymbiont *Spiroplasma*, named *sDis*, which also induces CI (Pollmann et al., 2022). This represents a novel phenotype for *Spiroplasma*. In the present work, we show that the occurrence of female offspring is reduced in crosses between BIR females and STU males, and can be rescued in crosses between tetracycline‐treated individuals. This suggests that *sDis* in STU is also able to induce CI in crosses with females of the BIR strain, which does not carry *Spiroplasma* (Appendix S1: Table A1). Among the barriers between these strains, CI was by far the strongest, albeit unilateral. It is unclear if this could drive the separation of these strains along with slight sexual isolation and mark the initiation of speciation, because these barriers do not cause strong reproductive isolation, as demonstrated by a total isolation of −0.28 and 0.14. Unidirectional CI is generally considered to be insufficient to drive speciation by itself because it is often incomplete, transmission is imperfect, and it can only be a barrier in one direction (Hurst & Schilthuizen, 1998; Telschow et al., 2007; Wade, 2001). However, it can represent the first and only barrier when acting bidirectionally as in *N. longicornis* and *N. giraulti* (Bordenstein et al., 2001), or in conjunction with other barriers as in *Encarsia*, *Drosophila* (Gebiola et al., 2016; Shoemaker et al., 1999), and between Clade A and Clade C of *L. distinguendus* where unidirectional CI is only one of several barriers (König, Zundel, et al., 2019).

### 4.6 Other postzygotic barriers

Behavioural sterility in hybrid females and a reduction in fertility of hybrid males were found between CAN (Clade B) and STU (Clade A), which are separated by 7.2% in COI, as well as in the more divergent strain pairs of BIR (Clade A) × SAT (Clade C) and CAN (Clade B) × PFO (Clade C) respectively. Apparently, they are the next barriers to emerge after sexual isolation and CI. Nothing is known about behavioural sterility of hybrid females in *L. distinguendus*. In *Nasonia*, significant behavioural sterility was only observed between *N. vitripennis* and *N. longicornis* (Beukeboom et al., 2015), but not between closer related species (Raychoudhury et al., 2010). A reduced fertility in hybrid males between CAN and STU was examined more closely by investigating sperm. This revealed a unidirectional reduction in sperm numbers in seminal vesicles of F2 hybrid male offspring, but no differences in the amount of sperm transferred to the spermathecae of females between hybrid and non‐hybrid males. Thus, it is unlikely that less sperm transferred to the females caused the reduction of female offspring of hybrid males. In *Nasonia*, hybrid physiological sterility has been linked to cytonuclear incompatibilities with dominance effects (Beukeboom et al., 2015; Koevoets et al., 2012), with negative effects depending on the ploidy level, rather than sex (Beukeboom et al., 2015). However, unlike in *Lariophagus*, neither sperm motility nor the production of females were impaired in *Nasonia* (Clark et al., 2010), suggesting different kinds of sperm impairment in both genera.

The last barriers to appear were identified between the strain combinations with the highest COI differences, BIR × SAT and CAN × PFO, respectively. They consisted of inviability, behavioural sterility and physiological sterility of hybrid males, and unidirectional reduction in the fertility of hybrid females, with the latter occurring only in the strain pair CAN × PFO. Therefore, these last barriers mostly affected hybrid males but not hybrid females. This points to Haldane's rule (Haldane, 1922), which states that postzygotic barriers, that is, negative effects on hybrids, appear first in the heterogametic sex. So far, numerous taxa have been found to obey Haldane's rule (see recent reviews by Delph & Demuth, 2016; Schilthuizen et al., 2011), making it an evolutionary pattern that seems to be almost universal, at least in the animal kingdom. However, these studies have mostly focused on diploid organisms with chromosomal sex determination, while haplodiploid organisms have been largely neglected. A recent review on Haldane's rule does not mention haplodiploids at all (Delph & Demuth, 2016). Phillips and Edmands (2012) hypothesize that because postzygotic isolation evolves more slowly in taxa with small X‐chromosomes as compared to taxa with large X‐chromosomes (Turelli & Begun, 1997), postzygotic isolation should evolve even slower in taxa without heteromorphic sex chromosomes like haplodiploids. In support of this idea, a meta‐analysis done by Lima (2014) found that taxa without sex chromosomes evolve lower levels of postzygotic isolation at a similar level of genetic divergence than taxa with sex chromosomes. However, Lima did not include taxa with haplodiploid sex determination either (Lima, 2014). In contrast to these ideas, Breeuwer and Werren (1995) and Koevoets and Beukeboom (2009) pointed to the fact that the basic mechanism of Haldane's rule, the stronger effect of DM incompatibilities on the heterogametic sex, also applies to males of haplodiploids, where the whole genome is hemizygous. This hypothesis is supported by our observation that intrinsic postzygotic barriers in *L. distinguendus* mostly affected hybrid males, but not hybrid females.

## 5 CONCLUSION

We investigated the cryptic diversity within the *L. distinguendus* species complex, a parasitoid which also occurs in human households, by inferring phylogenetic trees based on the COI gene and five nuclear genes as well as crossing experiments. We were able to confirm our previous results that strains collected on drugstore beetles and strains from granary weevils belong to different, albeit undescribed, species according to the BSC (König, Zundel, et al., 2019). In addition, we identified a third Clade, which also was collected on drugstore beetles and which can be considered a separate species based on the reproductive isolation from the other two species. Remarkably, although many of our strains were collected from the same area and with the same hosts as baits, they were genetically distinct enough as to belong to different Clades. This discovery highlights that cryptic biodiversity also exists in close proximity to humans but remains largely undetected.

The crossing experiments with a variety of strains along a gradient of relatedness indicate that reproductive isolation in *L. distinguendus* might have evolved from weak and unilateral sexual isolation, over behavioural sterility in hybrid females and reduced fertility of hybrid males, to strong sexual isolation and strong intrinsic postzygotic isolation (inviability, behavioural sterility, physiological sterility) affecting hybrid males. This supports the hypothesis that Haldane's rule also applies to Hymenoptera. In addition, the finding of CI caused by the endosymbiotic bacterium *Spiroplasma* between two closely related strains raises the question if speciation in *L. distinguendus* might be initiated by bacterial infestation, similar to the related *Nasonia*. Finally, barcoding by itself was found not to be suitable for species delimitation in *L. distinguendus*. It results in the separation of strains which are not reproductively isolated according to the BSC. Therefore, we suggest using data on reproductive isolation from crossing experiments with suitable candidate species for calibration to determine taxon‐specific thresholds that can then be used for species delimitation. Thereby, we should be able to reconcile the BSC, which is employed by most scientists studying ecology and evolution, and the species concepts based on molecular and morphological data, which are used by most taxonomists for species delimitation and species description.

## AUTHOR CONTRIBUTIONS

**Marie Pollmann:** Conceptualization (equal); data curation (lead); formal analysis (lead); funding acquisition (equal); investigation (lead); methodology (equal); project administration (supporting); supervision (equal); validation (equal); visualization (lead); writing – original draft (lead); writing – review and editing (lead). **Denise Kuhn:** Investigation (equal); methodology (equal). **Christian König:** Investigation (supporting); methodology (supporting). **Irmela Homolka:** Formal analysis (supporting); investigation (equal). **Sina Paschke:** Investigation (equal). **Ronja Reinisch:** Investigation (equal). **Anna Schmidt:** Investigation (equal). **Noa Schwabe:** Investigation (equal). **Justus Weber:** Investigation (equal). **Yuval Gottlieb:** Methodology (equal); resources (equal). **Johannes Luitpold Maria Steidle:** Conceptualization (equal); data curation (equal); formal analysis (equal); funding acquisition (lead); methodology (equal); project administration (lead); resources (lead); visualization (supporting); writing – original draft (supporting); writing – review and editing (supporting).

## CONFLICT OF INTEREST STATEMENT

The authors declare no competing interests.

## Supporting information


Appendix S1

## Data Availability

DNA sequences: GenBank, accession numbers are listed in Appendix S1: Table A5. Data from crossing experiments have been deposited on Dryad: https://doi.org/10.5061/dryad.sf7m0cgc3.
