# Supplementary material for: New species based on the biological species concept within the complex of Lariophagus distinguendus (Hymenoptera, Chalcidoidea, Pteromalidae), a parasitoid of household pests
Source: Ecol Evol. 2023 Sep 13;13(9):e10524. doi: 10.1002/ece3.10524 (PMC10500055; doi:10.1002/ece3.10524)
Supplement: Supplementary file 1 — Appendix S1 [file ECE3-13-e10524-s001.docx]

**Appendix**

Testing for endosymbionts

All strains were tested for the endosymbionts *Wolbachia* and *Spiroplasma*, which had been detected within *L. distinguendus* strains before (K. König *et al.*, 2019; Pollmann *et al.*, 2022). To that end, they were submitted to PCR amplification with the specific primer pairs W-Specf (5’- CAT ACC TAT TCG AAG GGA TAG-3‘) and W-Specr (5’- AGC TTC GAG TGA AAC CAA TTC-3’) (Werren and Windsor, 2000), with 95 °C for 2 minutes, 32 cycles of 94 °C for 30 seconds, 49 °C for 45 seconds, and 72 °C for 1 minute, followed by 72 °C for 1 minute, or *wsp* 81 F (5’-TGG TCC AAT AAG TGA TGA AGA AAC-3’) / *wsp* 691 R (5’-AAA AAT TAA ACG CTA CTC CA-3’) (Braig *et al.*, 1998), with 95 °C for 2 minutes, 35 cycles of 92 °C for 30 seconds, 58 °C for 30 seconds, and 72 °C for 30 seconds, followed by 72 °C for 5 minutes for *Wolbachia* and ApDnaAF1 (5’-ATT CTT CAG TAA AAA TGC TTG GA-3’) and ApDnaAR1 (5’-ACA CAT TTA CTT CAT GCT ATT GA-3’) (Fukatsu *et al.*, 2001), with 95 °C for 4 minutes, 35 cycles of 95 °C for 30 seconds and 55 °C for 30 seconds, and a final elongation at 72 °C for 1 minute, for *Spiroplasma*, respectively. Each PCR was conducted in 12.5 µl ROTI®Pol TaqS Red-Mix (2x) (Carl Roth GmbH + Co. KG, Karlsruhe, Germany), 1 µl of each primer, and 9.5 µl double distilled water per 1 µl template, with 5 µl Promega 5X Green GoTaq® Reaction Buffer, 2.5 µl 10mM dNTPs (Promega, Madison WI, USA), 1 µl of each primer, 14.3 µl double distilled water and 0.2 µl Promega GoTaq® G2 DNA Polymerase (Promega, Madison WI, USA) per 1 µl sample (or 2 µl sample with the amount of double distilled water reduced to 13.3 µl accordingly) or with 20 µl Promega GoTaq®Green Master Mix 2X (Promega, Madison WI, USA), 4 µl of each primer and 10 µl of double distilled water per 2 µl sample. A Techne® Prime thermal cycler (Cole-Parmer, Stone, UK), a Biometra TGradient 96 Thermocycler (Analytik Jena AG, Jena, Germany) or a Biometra professional Basic Thermocycler (Analytik Jena AG, Jena, Germany) were used.

Gel electrophoresis was conducted on a 1-2 % agarose gel with either 5 µl of peqgreen (VWR International GmbH, Darmstadt, Germany) or 5 µl ROTI®-GelStain (Carl Roth GmbH + Co. KG, Karlsruhe, Germany) per 100 ml as DNA markers and 10 µl of an equimolar 100 bp DNA ladder (Carl Roth GmbH + Co. KG, Karlsruhe, Germany) or Norgen LowRanger 100 bp DNA (Norgen Biotek Corp., Canada) as ladders. 5 to 10 µl per PCR product were transferred to the gel for visualization. PCR results were checked by sending the PCR products to Microsynth Seqlab (Göttingen, Germany) or Macrogen Europe (Amsterdam, The Netherlands) for Sanger sequencing.

Table A1: Status of infection with *Spiroplasma* and *Wolbachia* for all featured *L. distinguendus* strains.

| Strain | Infection status |
| --- | --- |
| dbBIR-D1 | None |
| dbBIR-D2 | *Spiroplasma* |
| dbBIR-D3 | *Spiroplasma* |
| dbBIR-D4 | None |
| dbBRU-D1 | *Spiroplasma* |
| gwBYG-DK1 | *Wolbachia* |
| dbCAN-D1 | *Spiroplasma* |
| dbCAN-D2 | *Spiroplasma* |
| dbFRI-D1 | None |
| dbLUD-D1 | *Spiroplasma* |
| dbOBE-D1 | *Spiroplasma* |
| dbOST-D1 | None |
| gwPFO-D1^a^ | *Wolbachia, Spiroplasma* |
| dbPLI-D2 | *Spiroplasma* |
| dbRAV-D1 | *Spiroplasma* |
| gwSAC-D1 | *Wolbachia* |
| gwSAT-D1^a^ | *Wolbachia* |
| gwSIL-D1 | *Wolbachia* |
| gwSLO-GB1^a^ | *Wolbachia* |
| dbSTU-D1^b^ | *Spiroplasma* |
| dbSTU-D3 | *Spiroplasma* |
| gwSWD-D1 | *Wolbachia, Spiroplasma* |
| dbVAI-D1 | *Spiroplasma* |
| dbVAI-D2 | *Spiroplasma* |
| dbVAI-D3 | *Spiroplasma* |
| dbVAI-D4 | *Spiroplasma* |
| dbWAG-N1 | *Wolbachia* |
| dbWAN-D1 | *Spiroplasma* |

^a^(K. König *et al.*, 2019); ^b^(Pollmann *et al.*, 2022)

Table A2: Numbers of overall characters and character types for all genes.

| Gene | Characters (of aligned sequences) | Constant characters | Parsimony-informative characters | Singletons |
| --- | --- | --- | --- | --- |
| COI | 652 | 466 | 129 | 57 |
| CAD | 550 | 452 | 5 | 93 |
| ITS2 | 449 | 359 | 8 | 82 |
| LOC 100123206 | 432 | 378 | 3 | 51 |
| LOC 100123909 | 511 | 449 | 6 | 56 |
| LOC 100117339 | 550 | 454 | 4 | 92 |
| Sum of all genes | 3144 | 2558 | 155 | 401 |

Table A3: Partitions and best-fit substitution models for all genes.

| Partitions | | Substitution models |
| --- | --- | --- |
| COI | |  |
| Subset 1 | 1^st^ codon position | TrN+F+G4 |
| Subset 2 | 2^nd^ codon position | TrN+F+G4 |
| Subset 3 | 3^rd^ codon position | F81+F |
| Concatenation of nuclear genes | |  |
| Subset 1 | CAD Exon 3^rd^ codon position | K2P |
| Subset 2 | CAD Exon 1^st^ codon position  LOC 100123206 Exon 1^st^ codon position  LOC 100123909 Exon 1^st^ codon position  LOC 100117339 Exon 1^st^ codon position | F81+F |
| Subset 3 | CAD Exon 2^nd^ codon position  LOC 100123206 Exon 2^nd^ codon position  LOC 100123909 Exon 2^nd^ codon position  LOC 100117339 Exon 2^nd^ codon position | F81+F |
| Subset 4 | CAD Intron  LOC 100123206 Intron1  LOC 100123206 Intron2  LOC 100123909 Intron1  LOC 100117339 Intron1  LOC 100117339 Intron2  LOC 100117339 Intron3 | TPM3+F |
| Subset 5 | LOC 100123206 Exon 3^rd^ codon position  LOC 100123909 Exon 3^rd^ codon position  LOC 100123909 Intron2  LOC 100117339 Exon 3^rd^ codon position | HKY+F |
| Subset 6 | ITS2 | JC+I |

Table A4: Accession numbers of sequences retrieved from GenBank used in this study. Sequences in bold were generated by (König *et al.*, 2015).

| Strain | COI  (barcode segment) | ITS2 | CAD | LOC100123206 | LOC100123909 | LOC100117339 |
| --- | --- | --- | --- | --- | --- | --- |
| dbBIR-D1 | - | **KJ923919** | **KJ867408** | **KJ923863** | **KJ923883** | **KJ923901** |
| gwBYG-DK1 | - | **KJ923905** | **KJ867400** | **KJ923856** | **KJ923875** | **KJ923893** |
| gwPFO-D1 | - | **KJ923903** | **KJ867392** | **KJ923849** | **KJ923867** | **KJ923885** |
| dbRAV-D1 | - | **KJ923913** | **KJ867404** | **KJ923859** | **KJ923879** | **KJ923897** |
| gwSAC-D1 | - | **KJ923912** | **KJ867396** | **KJ923852** | **KJ923871** | **KJ923889** |
| gwSAT-D1 | - | **KJ923908** | **KJ867394** | **KJ923851** | **KJ923869** | **KJ923887** |
| gwSLO-GB1 | - | **KJ923910** | **KJ867398** | **KJ923854** | **KJ923873** | **KJ923891** |
| dbSTU-D1 | - | **KJ923915** | **KJ867402** | **KJ923866** | **KJ923877** | **KJ923895** |
| dbWAG-N1 | - | **KJ923917** | **KJ867406** | **KJ923861** | **KJ923881** | **KJ923899** |
| *Nasonia vitripennis* | EU746537 | **KJ923923** | KC213163 | **KJ923921** | XM_001607631 | **KJ923922** |
| *Eupelmus confusus* | KJ018429 | - | - | - | - | - |

Table A5: GenBank accession numbers of all sequences generated in this study.

| Strain | Specimen ID | COI  (barcode segment) | ITS2 | CAD | LOC100123206 | LOC100123909 | LOC100117339 |
| --- | --- | --- | --- | --- | --- | --- | --- |
| dbBIR-D1 | UHdbBIR-D1 I | OQ933721 | **-** | **-** | **-** | **-** | **-** |
| dbBIR-D2 | UHdbBIR-D2 I | OQ933722 | OQ938963 | OQ939843 | OQ939881 | OQ939900 | OQ939862 |
| dbBIR-D3 | UHdbBIR-D3 I | OQ933723 | OQ938964 | OQ939844 | OQ939882 | OQ939901 | OQ939863 |
| dbBIR-D4 | UHdbBIR-D4 I | OQ933724 | OQ938965 | OQ939845 | OQ939883 | OQ939902 | OQ939864 |
| dbBRU-D1 | UHdbBRU-D1 I | OQ933725 | OQ938966 | OQ939846 | OQ939884 | OQ939903 | OQ939865 |
| gwBYG-DK1 | UHgwBYG-DK1 I | OQ933726 | **-** | **-** | **-** | **-** | **-** |
| dbCAN-D1 | UHdbCAN-D1 I | OQ933727 | OQ938967 | OQ939847 | OQ939885 | OQ939904 | OQ939866 |
| dbCAN-D2 | UHdbCAN-D2 I | OQ933728 | OQ938968 | OQ939848 | OQ939886 | OQ939905 | OQ939867 |
| dbFRI-D1 | UHdbFRI-D1 I | OQ933729 | OQ938969 | OQ939849 | OQ939887 | OQ939906 | OQ939868 |
| dbLUD-D1 | UHdbLUD-D1 I | OQ933730 | OQ938970 | OQ939850 | OQ939888 | OQ939907 | OQ939869 |
| dbOBE-D1 | UHdbOBE-D1 I | OQ933731 | OQ938971 | OQ939851 | OQ939889 | OQ939908 | OQ939870 |
| dbOST-D1 | UHdbOST-D1 I | OQ933732 | OQ938972 | OQ939852 | OQ939890 | OQ939909 | OQ939871 |
| gwPFO-D1 | UHgwPFO-D1 I | OQ933733 | **-** | **-** | **-** | **-** | **-** |
| dbPLI-D2 | UHdbPLI-D2 I | OQ933734 | OQ938973 | OQ939853 | OQ939891 | OQ939910 | OQ939872 |
| dbRAV-D1 | UHdbRAV-D1 I | OQ933735 | **-** | **-** | **-** | **-** | **-** |
| gwSAC-D1 | UHgwSAC-D1 I | OQ933736 | **-** | **-** | **-** | **-** | **-** |
| gwSAT-D1 | UHgwSAT-D1 I | OQ933737 | **-** | **-** | **-** | **-** | **-** |
| gwSIL-D1 | UHgwSIL-D1 I | OQ933738 | OQ938974 | OQ939854 | OQ939892 | OQ939917 | OQ939873 |
| gwSLO-GB1 | UHgwSLO-GB1 I | OQ933739 | **-** | **-** | **-** | **-** | **-** |
| dbSTU-D1 | UHdbSTU-D1 I | OQ933740 | **-** | **-** | **-** | **-** | **-** |
| dbSTU-D3 | UHdbSTU-D3 I | OQ933741 | OQ938975 | OQ939855 | OQ939893 | OQ939911 | OQ939874 |
| gwSWD-D1 | UHgwSWD-D1 I | OQ933742 | OQ938976 | OQ939856 | OQ939894 | OQ939918 | OQ939875 |
| dbVAI-D1 | UHdbVAI-D1 I | OQ933743 | OQ938977 | OQ939857 | OQ939895 | OQ939912 | OQ939876 |
| dbVAI-D2 | UHdbVAI-D2 I | OQ933744 | OQ938978 | OQ939858 | OQ939896 | OQ939913 | OQ939877 |
| dbVAI-D3 | UHdbVAI-D3 I | OQ933745 | OQ938979 | OQ939859 | OQ939897 | OQ939914 | OQ939878 |
| dbVAI-D4 | UHdbVAI-D4 I | OQ933746 | OQ938980 | OQ939860 | OQ939898 | OQ939915 | OQ939879 |
| dbWAG-N1 | UHdbWAG-N1 I | OQ933747 | **-** | **-** | **-** | **-** | **-** |
| dbWAN-D1 | UHdbWAN-D1 I | OQ933748 | OQ938981 | OQ939861 | OQ939899 | OQ939916 | OQ939880 |

Table A6: Uncorrected p-distances of the barcode segment of COI for all *L. distinguendus* strains used for crossing experiments and mean p-distances between the clades based on all strains. Distances for strain combinations in bold.

| Clade |  |  |  |  |  |  | Clade | |
| --- | --- | --- | --- | --- | --- | --- | --- | --- |
|  | strain | STU | OST | BIR | CAN | PFO | A | B |
| A | STU |  |  |  |  |  |  |  |
|  | OST | 0.022 |  |  |  |  |  |  |
|  | BIR | **0.028** | **0.017** |  |  |  |  |  |
| B | CAN | **0.072** | 0.068 | 0.074 |  |  | 0.0717 |  |
| C | PFO | 0.131 | 0.132 | 0.140 | **0.140** |  | 0.1328 | 0.1422 |
|  | SAT | 0.126 | 0.132 | **0.139** | 0.143 | 0.031 |  |  |

**Statistical analyses**

**Sexual isolation**

Table A7: Comparison of the occurrence of copulation in crosses between males and females of the *L. distinguendus* strains BIR and OST. Comparisons were made using the 2 x 2 Fisher’s Exact test for Count Data.

|  |  | *p* value |
| --- | --- | --- |
| BIR ♀ x BIR ♂ | BIR ♀ x OST ♂ | 0.02141 * |
| OST ♀ x BIR ♂ | OST ♀ x OST ♂ | 0.03551 * |

* *p <* 0.05

Table A8: Comparison of the occurrence of copulation in crosses between males and females of the *L. distinguendus* strains BIR and STU. A Pearson's Chi-squared test was conducted for the comparisons.

|  |  | *p* value |
| --- | --- | --- |
| BIR ♀ x BIR ♂ | BIR ♀ x STU ♂ | 0.512 n.s. |
| STU ♀ x BIR ♂ | STU ♀ x STU ♂ | 0.04382 * |

n.s. *p* > 0.05, * *p* < 0.05

Table A9: Comparison of the occurrence of copulation in crosses between males and females of the *L. distinguendus* strains CAN and STU. Comparisons were made using the 2 x 2 Fisher’s Exact test for Count Data.

|  |  | *p* value |
| --- | --- | --- |
| CAN ♀ x CAN ♂ | CAN ♀ x STU ♂ | 0.7411 |
| STU ♀ x CAN ♂ | STU ♀ x STU ♂ | 0.00953 ** |

n.s. *p* > 0.05, ** *p* < 0.01

Table A10: Comparison of the occurrence of copulation in crosses between males and females of the *L. distinguendus* strains BIR and SAT. Comparisons were made using the 2 x 2 Fisher’s Exact test for Count Data.

|  |  | *p* value |
| --- | --- | --- |
| BIR ♀ x BIR ♂ | BIR ♀ x SAT ♂ | 0.0004359 *** |
| SAT ♀ x BIR ♂ | SAT ♀ x SAT ♂ | 2.444e-07 *** |

*** *p* < 0.001

Table A11: Comparison of the occurrence of copulation in crosses between males and females of the *L. distinguendus* strains CAN and PFO. Comparisons were made using the 2 x 2 Fisher’s Exact test for Count Data.

|  |  | *p* value |
| --- | --- | --- |
| CAN ♀ x CAN ♂ | CAN ♀ x PFO ♂ | 7.709e-07 *** |
| PFO ♀ x CAN ♂ | PFO ♀ x PFO ♂ | 3.358e-06 *** |

*** *p* < 0.001

**Female inviability**

Table A12: Results of the linear model (number of female offspring ~ crossing) comparing the number of F1 female offspring in intra- and interstrain crossings between males and females of the *L. distinguendus* strains BIR and OST and the subsequent Tukey test for multiple comparisons. The reference contained in the intercept is the crossing BIR ♀ x BIR ♂.

|  | | Estimate | | Std. Error | | t value | | Pr(>\|t\|) | |
| --- | --- | --- | --- | --- | --- | --- | --- | --- | --- |
| Intercept | | 31.348 | | 3.717 | | 8.435 | | 3.88e-13*** | |
| BIR ♀ x OST ♂ | | 2.075 | | 5.102 | | 0.407 | | 0.6851 | |
| OST ♀ x BIR ♂ | | 10.152 | | 5.201 | | 1.952 | | 0.0539 | |
| OST ♀ x OST ♂ | | 3.012 | | 5.150 | | 0.585 | | 0.5600 | |
| Tukey test for multiple comparisons | | | | | | | | | |
|  |  | | Estimate | | Std. Error | | z value | | Pr(>\|t\|) |
| BIR ♀ x BIR ♂ | BIR ♀ x OST ♂ | | 2.0753 | | 5.1021 | | 0.407 | | 0.977 |
|  | OST ♀ x BIR ♂ | | 10.1522 | | 5.2010 | | 1.952 | | 0.214 |
|  | OST ♀ x OST ♂ | | 3.0122 | | 5.1498 | | 0.585 | | 0.936 |
| BIR ♀ x OST ♂ | OST ♀ x BIR ♂ | | 8.0769 | | 5.0454 | | 1.601 | | 0.383 |
|  | OST ♀ x OST ♂ | | 0.9369 | | 4.9927 | | 0.188 | | 0.998 |
| OST ♀ x BIR ♂ | OST ♀ x OST ♂ | | -7.1400 | | 5.0936 | | -1.402 | | 0.501 |

n.s. *p* > 0.05, *** *p* < 0.001

Table A13: Results of the generalized linear model (number of female offspring ~ crossing, family= quasipoisson) comparing the number of F1 female offspring in intra- and interstrain crossings between males and females of the *L. distinguendus* strains BIR and STU and the subsequent Tukey test for multiple comparisons. The reference contained in the intercept is the crossing BIR ♀ x BIR ♂.

|  | | Estimate | | Std. Error | | t value | | Pr(>\|t\|) | |
| --- | --- | --- | --- | --- | --- | --- | --- | --- | --- |
| Intercept | | 3.10837 | | 0.10847 | | 28.657 | | <2e-16 *** | |
| BIR ♀ x STU ♂ | | 0.04459 | | 0.15371 | | 0.290 | | 0.772 | |
| STU ♀ x BIR ♂ | | 0.18931 | | 0.14580 | | 1.298 | | 0.196 | |
| STU ♀ x STU ♂ | | 0.12265 | | 0.15179 | | 0.808 | | 0.420 | |
| Tukey test for multiple comparisons | | | | | | | | | |
|  |  | | Estimate | | Std. Error | | z value | | Pr(>\|z) |
| BIR ♀ x BIR ♂ | BIR ♀ x STU ♂ | | 0.04459 | | 0.15371 | | 0.290 | | 0.991 |
|  | STU ♀ x BIR ♂ | | 0.18931 | | 0.14580 | | 1.298 | | 0.564 |
|  | STU ♀ x STU ♂ | | 0.12265 | | 0.15179 | | 0.808 | | 0.851 |
| BIR ♀ x STU ♂ | STU ♀ x BIR ♂ | | 0.14472 | | 0.14613 | | 0.990 | | 0.755 |
|  | STU ♀ x STU ♂ | | 0.07806 | | 0.15210 | | 0.513 | | 0.956 |
| STU ♀ x BIR ♂ | STU ♀ x STU ♂ | | -0.06666 | | 0.14411 | | -0.463 | | 0.967 |

n.s. *p* > 0.05, *** *p* < 0.001

Table A14: Results of the linear model (number of female offspring ~ crossing) comparing the number of F1 female offspring in intra- and interstrain crossings between males and females of the *L. distinguendus* strains CAN and STU and the subsequent Tukey test for multiple comparisons. The reference contained in the intercept is the crossing CAN ♀ x CAN ♂.

|  | | Estimate | | Std. Error | | t value | | Pr(>\|t\|) | |
| --- | --- | --- | --- | --- | --- | --- | --- | --- | --- |
| Intercept | | 31.474 | | 4.044 | | 7.782 | | 4.6e-11 *** | |
| CAN ♀ x STU ♂ | | -4.363 | | 5.798 | | -0.752 | | 0.45434 | |
| STU ♀ x CAN ♂ | | 14.726 | | 5.647 | | 2.608 | | 0.01114 * | |
| STU ♀ x STU ♂ | | 19.820 | | 5.885 | | 3.368 | | 0.00123 ** | |
| Tukey test for multiple comparisons | | | | | | | | | |
|  |  | | Estimate | | Std. Error | | z value | | Pr(>\|t) |
| CAN ♀ x CAN ♂ | CAN ♀ x STU ♂ | | -4.363 | | 5.798 | | -0.752 | | 0.87529 |
|  | STU ♀ x CAN ♂ | | 14.726 | | 5.647 | | 2.608 | | 0.05296 |
|  | STU ♀ x STU ♂ | | 19.820 | | 5.885 | | 3.368 | | 0.00699 ** |
| CAN ♀ x STU ♂ | STU ♀ x CAN ♂ | | 19.089 | | 5.727 | | 3.333 | | 0.00737 ** |
|  | STU ♀ x STU ♂ | | 24.183 | | 5.962 | | 4.056 | | < 0.001 *** |
| STU ♀ x CAN ♂ | STU ♀ x STU ♂ | | 5.094 | | 5.815 | | 0.876 | | 0.81719 |

n.s. *p* > 0.05, * *p* < 0.05, ** *p* < 0.01, *** *p* < 0.001

Table A15: Results of the generalized linear model (number of female offspring ~ crossing, family= quasipoisson) comparing the number of F1 female offspring in intra- and interstrain crossings between males and females of the *L. distinguendus* strains BIR and SAT and the subsequent Tukey test for multiple comparisons. The reference contained in the intercept is the crossing BIR ♀ x BIR ♂.

|  | | Estimate | | Std. Error | | t value | | Pr(>\|t\|) | |
| --- | --- | --- | --- | --- | --- | --- | --- | --- | --- |
| Intercept | | 3.84791 | | 0.11181 | | 34.416 | | < 2e-16 *** | |
| BIR ♀ x SAT ♂ | | -0.75686 | | 0.51540 | | -1.469 | | 0.148 | |
| SAT ♀ x BIR ♂ | | 0.16170 | | 0.15852 | | 1.020 | | 0.312 | |
| SAT ♀ x SAT ♂ | | -0.02961 | | 0.15930 | | -0.186 | | 0.853 | |
| Tukey test for multiple comparisons | | | | | | | | | |
|  |  | | Estimate | | Std. Error | | z value | | Pr(>\|z) |
| BIR ♀ x BIR ♂ | BIR ♀ x SAT ♂ | | -0.75686 | | 0.51540 | | -1.469 | | 0.428 |
|  | SAT ♀ x BIR ♂ | | 0.16170 | | 0.15852 | | 1.020 | | 0.717 |
|  | SAT ♀ x SAT ♂ | | -0.02961 | | 0.15930 | | -0.186 | | 0.997 |
| BIR ♀ x SAT ♂ | SAT ♀ x BIR ♂ | | 0.91856 | | 0.51552 | | 1.782 | | 0.257 |
|  | SAT ♀ x SAT ♂ | | 0.72725 | | 0.51576 | | 1.410 | | 0.464 |
| SAT ♀ x BIR ♂ | SAT ♀ x SAT ♂ | | -0.19131 | | 0.15970 | | -1.198 | | 0.602 |

n.s. *p* > 0.05, *** *p* < 0.001

Table A16: Results of the linear model (number of female offspring ~ crossing) comparing the number of F1 female offspring in intra- and interstrain crossings between males and females of the *L. distinguendus* strains CAN and PFO and the subsequent Tukey test for multiple comparisons. The reference contained in the intercept is the crossing CAN ♀ x CAN ♂.

|  | | Estimate | | Std. Error | | t value | | Pr(>\|t\|) | |
| --- | --- | --- | --- | --- | --- | --- | --- | --- | --- |
| Intercept | | 41.421 | | 4.583 | | 9.038 | | 2.55e-12 *** | |
| CAN ♀ x PFO ♂ | | -40.421 | | 20.495 | | -1.972 | | 0.0538 | |
| PFO ♀ x CAN ♂ | | -9.199 | | 6.570 | | -1.400 | | 0.1673 | |
| PFO ♀ x PFO ♂ | | -5.474 | | 6.481 | | -0.845 | | 0.4021 | |
| Tukey test for multiple comparisons | | | | | | | | | |
|  |  | | Estimate | | Std. Error | | t value | | Pr(>\|t\|) |
| CAN ♀ x CAN ♂ | CAN ♀ x PFO ♂ | | -40.421 | | 20.495 | | -1.972 | | 0.191 |
|  | PFO ♀ x CAN ♂ | | -9.199 | | 6.570 | | -1.400 | | 0.478 |
|  | PFO ♀ x PFO ♂ | | -5.474 | | 6.481 | | -0.845 | | 0.818 |
| CAN ♀ x PFO ♂ | PFO ♀ x CAN ♂ | | 31.222 | | 20.523 | | 1.521 | | 0.405 |
|  | PFO ♀ x PFO ♂ | | 34.947 | | 20.495 | | 1.705 | | 0.306 |
| PFO ♀ x CAN ♂ | PFO ♀ x PFO ♂ | | 3.725 | | 6.570 | | 0.567 | | 0.936 |

**Female behavioural sterility**

Table A17: Comparison of the occurrence of copulation in crosses between males and F1 female offspring of the *L. distinguendus* strains BIR and OST. Comparisons were made using the 2 x 2 Fisher’s Exact test for Count Data.

|  |  | *p* value |
| --- | --- | --- |
| BIR ♀ x BIR ♂ | BIR ♀ x OST ♂ | 0.1299 n.s. |
| OST ♀ x BIR ♂ | OST ♀ x OST ♂ | 0.749 n.s. |

n.s. *p* > 0.05

Table A18: Comparison of the occurrence of copulation in crosses between males and F1 female offspring of the *L. distinguendus* strains BIR and STU. A Pearson's Chi-squared test was conducted for the comparison.

|  |  | *p* value |
| --- | --- | --- |
| BIR ♀ x BIR ♂ | BIR ♀ x STU ♂ | 0.9334 n.s. |
| STU ♀ x BIR ♂ | STU ♀ x STU ♂ | 0.9018 n.s. |

n.s. *p* > 0.05

Table A19: Comparison of the occurrence of copulation in crosses between males and F1 female offspring of the *L. distinguendus* strains CAN and STU. Comparisons were made using the 2 x 2 Fisher’s Exact test for Count Data.

|  |  | *p* value |
| --- | --- | --- |
| CAN ♀ x CAN ♂ | CAN ♀ x STU ♂ | 0.04091 * |
| STU ♀ x CAN ♂ | STU ♀ x STU ♂ | 0.0003284 *** |

* *p* < 0.05, *** *p* < 0.001

**Female physiological sterility**

Table A20: Comparison of the occurrence of F2 offspring in crosses between males and F1 female offspring of the *L. distinguendus* strains BIR and OST. Comparisons were made using the 2 x 2 Fisher’s Exact test for Count Data.

|  |  | *p* value |
| --- | --- | --- |
| BIR ♀ x BIR ♂ | BIR ♀ x OST ♂ | 1 n.s. |
| OST ♀ x BIR ♂ | OST ♀ x OST ♂ | 0.4923 n.s. |

n.s. *p* > 0.05

Table A21: Comparison of the occurrence of F2 offspring in crosses between males and F1 female offspring of the *L. distinguendus* strains BIR and STU. Comparisons were made using the 2 x 2 Fisher’s Exact test for Count Data.

|  |  | *p* value |
| --- | --- | --- |
| BIR ♀ x BIR ♂ | BIR ♀ x STU ♂ | 1 n.s. |
| STU ♀ x BIR ♂ | STU ♀ x STU ♂ | 0.61 n.s. |

n.s. *p* > 0.05

Table A22: Comparison of the occurrence of F2 offspring in crosses between males and F1 female offspring of the *L. distinguendus* strains CAN and STU. Comparisons were made using the 2 x 2 Fisher’s Exact test for Count Data.

|  |  | *p* value |
| --- | --- | --- |
| CAN ♀ x CAN ♂ | CAN ♀ x STU ♂ | 1 n.s. |
| STU ♀ x CAN ♂ | STU ♀ x STU ♂ | 1 n.s. |

n.s. *p* > 0.05

Table A23: Comparison of the occurrence of F2 offspring in crosses between males and F1 female offspring of the *L. distinguendus* strains BIR and SAT. Comparisons were made using the 2 x 2 Fisher’s Exact test for Count Data.

|  |  | *p* value |
| --- | --- | --- |
| BIR ♀ x BIR ♂ | BIR ♀ x SAT ♂ | 1 n.s. |
| SAT ♀ x BIR ♂ | SAT ♀ x SAT ♂ | 0.106 n.s. |

n.s. *p* > 0.05

Table A24: Comparison of the occurrence of F2 offspring in crosses between males and F1 female offspring of the *L. distinguendus* strains CAN and PFO. Comparisons were made using the 2 x 2 Fisher’s Exact test for Count Data.

|  |  | *p* value |
| --- | --- | --- |
| CAN ♀ x CAN ♂ | CAN ♀ x PFO ♂ | N/A |
| PFO ♀ x CAN ♂ | PFO ♀ x PFO ♂ | 1 n.s. |

n.s. *p* > 0.05

**Female physiological reduced fertility**

Table A25: Results of the generalized linear model (total offspring number ~ crossing, family=quasipoisson) comparing the total number of F2 offspring of mated F1 female offspring of intra- and interstrain crossings between the *L. distinguendus* strains BIR and OST and wildtype F1 males and the subsequent Tukey test for multiple comparisons. The reference contained in the intercept is the crossing BIR ♀ x BIR ♂.

|  | | Estimate | | Std. Error | | t value | | Pr(>\|t\|) | |
| --- | --- | --- | --- | --- | --- | --- | --- | --- | --- |
| Intercept | | 3.89776 | | 0.08478 | | 45.978 | | <2e-16 *** | |
| BIR ♀ x OST ♂ | | 0.13147 | | 0.11731 | | 1.121 | | 0.2657 | |
| OST ♀ x BIR ♂ | | 0.20819 | | 0.11912 | | 1.748 | | 0.0843 | |
| OST ♀ x OST ♂ | | 0.14041 | | 0.12456 | | 1.127 | | 0.2629 | |
| Tukey test for multiple comparisons | | | | | | | | | |
|  |  | | Estimate | | Std. Error | | z value | | Pr(>\|t\|) |
| BIR ♀ x BIR ♂ | BIR ♀ x OST ♂ | | 0.13147 | | 0.13956 | | 0.942 | | 0.782 |
|  | OST ♀ x BIR ♂ | | 0.20819 | | 0.14455 | | 1.440 | | 0.474 |
|  | OST ♀ x OST ♂ | | 0.14041 | | 0.14905 | | 0.942 | | 0.782 |
| BIR ♀ x OST ♂ | OST ♀ x BIR ♂ | | 0.07672 | | 0.14561 | | 0.527 | | 0.953 |
|  | OST ♀ x OST ♂ | | 0.00894 | | 0.15008 | | 0.060 | | 1.000 |
| OST ♀ x BIR ♂ | OST ♀ x OST ♂ | | -0.06778 | | 0.15473 | | -0.438 | | 0.972 |

n.s. *p* > 0.05, *** *p* < 0.001

Table A26: Results of the generalized linear model (total offspring number ~ crossing, family=quasipoisson) comparing the total number of F2 offspring of mated F1 female offspring of intra- and interstrain crossings between the *L. distinguendus* strains BIR and STU and wildtype F1 males and the subsequent Tukey test for multiple comparisons. The reference contained in the intercept is the crossing BIR ♀ x BIR ♂.

|  | | Estimate | | Std. Error | | t value | | Pr(>\|t\|) | |
| --- | --- | --- | --- | --- | --- | --- | --- | --- | --- |
| Intercept | | 3.0875 | | 0.1038 | | 29.739 | | <2e-16 *** | |
| BIR ♀ x STU ♂ | | 0.2844 | | 0.1390 | | 2.046 | | 0.0426 * | |
| STU ♀ x BIR ♂ | | 0.1023 | | 0.1470 | | 0.696 | | 0.4875 | |
| STU ♀ x STU ♂ | | -0.0318 | | 0.1490 | | -0.213 | | 0.8313 | |
| Tukey test for multiple comparisons | | | | | | | | | |
|  |  | | Estimate | | Std. Error | | z value | | Pr(>\|z\|) |
| BIR ♀ x BIR ♂ | BIR ♀ x STU ♂ | | 0.2844 | | 0.1390 | | 2.046 | | 0.171 |
|  | STU ♀ x BIR ♂ | | 0.1023 | | 0.1470 | | 0.696 | | 0.899 |
|  | STU ♀ x STU ♂ | | -0.0318 | | 0.1490 | | -0.213 | | 0.997 |
| BIR ♀ x STU ♂ | STU ♀ x BIR ♂ | | -0.1821 | | 0.1393 | | -1.307 | | 0.558 |
|  | STU ♀ x STU ♂ | | -0.3162 | | 0.1413 | | -2.238 | | 0.113 |
| STU ♀ x BIR ♂ | STU ♀ x STU ♂ | | -0.1341 | | 0.1492 | | -0.899 | | 0.805 |

n.s. *p* > 0.05, * *p* < 0.05, *** *p* < 0.001

Table A27: Results of the linear model (total offspring number ~ crossing) comparing the total number of F2 offspring of mated F1 female offspring of intra- and interstrain crossings between the *L. distinguendus* strains CAN and STU and wildtype F1 males and the subsequent Tukey test for multiple comparisons. The reference contained in the intercept is the crossing CAN ♀ x CAN ♂.

|  | | Estimate | | Std. Error | | t value | | Pr(>\|t\|) | |
| --- | --- | --- | --- | --- | --- | --- | --- | --- | --- |
| Intercept | | 50.611 | | 3.971 | | 12.744 | | <2e-16 *** | |
| CAN ♀ x STU ♂ | | -12.729 | | 5.698 | | -2.234 | | 0.0286 * | |
| STU ♀ x CAN ♂ | | 3.889 | | 5.474 | | 0.710 | | 0.4798 | |
| STU ♀ x STU ♂ | | -1.661 | | 5.474 | | -0.303 | | 0.7624 | |
| Tukey test for multiple comparisons | | | | | | | | | |
|  |  | | Estimate | | Std. Error | | z value | | Pr(>\|t\|) |
| CAN ♀ x CAN ♂ | CAN ♀ x STU ♂ | | -12.729 | | 5.698 | | -2.234 | | 0.1241 |
|  | STU ♀ x CAN ♂ | | 3.889 | | 5.474 | | 0.710 | | 0.8926 |
|  | STU ♀ x STU ♂ | | -1.661 | | 5.474 | | -0.303 | | 0.9902 |
| CAN ♀ x STU ♂ | STU ♀ x CAN ♂ | | 16.618 | | 5.558 | | 2.990 | | 0.0198 * |
|  | STU ♀ x STU ♂ | | 11.068 | | 5.558 | | 1.991 | | 0.2010 |
| STU ♀ x CAN ♂ | STU ♀ x STU ♂ | | -5.550 | | 5.328 | | -1.042 | | 0.7256 |

n.s. *p* > 0.05, * *p* < 0.05, *** *p* < 0.001

Table A28: Results of the generalized linear model (total offspring number ~ crossing, family=quasipoisson) comparing the total number of F2 offspring of mated F1 female offspring of intra- and interstrain crossings between the *L. distinguendus* strains BIR and SAT and wildtype F1 males and the subsequent Tukey test for multiple comparisons. The reference contained in the intercept is the crossing BIR ♀ x BIR ♂.

|  | | Estimate | | Std. Error | | t value | | Pr(>\|t\|) | |
| --- | --- | --- | --- | --- | --- | --- | --- | --- | --- |
| Intercept | | 4.1207 | | 0.1030 | | 39.992 | | < 2e-16 *** | |
| BIR ♀ x SAT ♂ | | 0.1105 | | 0.1721 | | 0.642 | | 0.523068 | |
| SAT ♀ x BIR ♂ | | -0.6643 | | 0.1768 | | -3.758 | | 0.000377 *** | |
| SAT ♀ x SAT ♂ | | -0.6642 | | 0.1868 | | -3.556 | | 0.000721 *** | |
| Tukey test for multiple comparisons | | | | | | | | | |
|  |  | | Estimate | | Std. Error | | z value | | Pr(>\|z\|) |
| BIR ♀ x BIR ♂ | BIR ♀ x SAT ♂ | | 0.1105419 | | 0.1721275 | | 0.642 | | 0.91747 |
|  | SAT ♀ x BIR ♂ | | -0.6643452 | | 0.1767686 | | -3.758 | | < 0.001 *** |
|  | SAT ♀ x SAT ♂ | | -0.6641596 | | 0.1867706 | | -3.556 | | 0.00212 ** |
| BIR ♀ x SAT ♂ | SAT ♀ x BIR ♂ | | -0.7748871 | | 0.1991022 | | -3.892 | | < 0.001 *** |
|  | SAT ♀ x SAT ♂ | | -0.7747015 | | 0.2080332 | | -3.724 | | 0.00117 ** |
| SAT ♀ x BIR ♂ | SAT ♀ x SAT ♂ | | 0.0001855 | | 0.2118893 | | 0.001 | | 1.00000 |

n.s. *p* > 0.05, ** *p* < 0.01, *** *p* < 0.001

Table A29: Results of the linear model (total offspring number ~ crossing) comparing the total number of F2 offspring of mated F1 female offspring of intra- and interstrain crossings between the *L. distinguendus* strains CAN and PFO and wildtype F1 males and the subsequent Tukey test for multiple comparisons. The reference contained in the intercept is the crossing CAN ♀ x CAN ♂.

|  | | Estimate | | Std. Error | | t value | | Pr(>\|t\|) | |
| --- | --- | --- | --- | --- | --- | --- | --- | --- | --- |
| Intercept | | 88.650 | | 5.756 | | 15.400 | | < 2e-16 *** | |
| CAN ♀ x PFO ♂ | | N/A due to absence of mating in parental generation | | | | | | | |
| PFO ♀ x CAN ♂ | | -65.650 | | 8.364 | | -7.849 | | 2.2e-10 *** | |
| PFO ♀ x PFO ♂ | | -20.238 | | 8.492 | | -2.383 | | 0.0209 * | |
| Tukey test for multiple comparisons | | | | | | | | | |
|  |  | | Estimate | | Std. Error | | t value | | Pr(>\|t\|) |
| CAN ♀ x CAN ♂ | PFO ♀ x CAN ♂ | | -65.650 | | 8.364 | | -7.849 | | <0.001 *** |
|  | PFO ♀ x PFO ♂ | | -20.238 | | 8.492 | | -2.383 | | 0.0534 |
| PFO ♀ x CAN ♂ | PFO ♀ x PFO ♂ | | 45.412 | | 8.706 | | 5.216 | | <0.001 *** |

n.s. *p* > 0.05, * *p* < 0.05, *** *p* < 0.001

**Male inviability**

Table A30: Results of the generalized linear model (number of male offspring ~ crossing, family=quasipoisson) comparing the number of F2 male offspring of virgin F1 female offspring of intra- and interstrain crossings between the *L. distinguendus* strains BIR and OST and the subsequent Tukey test for multiple comparisons. The reference contained in the intercept is the crossing BIR ♀ x BIR ♂.

|  | | Estimate | | Std. Error | | t value | | Pr(>\|t\|) | |
| --- | --- | --- | --- | --- | --- | --- | --- | --- | --- |
| Intercept | | 3.95124 | | 0.08761 | | 45.102 | | <2e-16 *** | |
| BIR ♀ x OST ♂ | | 0.13849 | | 0.11289 | | 1.227 | | 0.223 | |
| OST ♀ x BIR ♂ | | 0.13173 | | 0.11385 | | 1.157 | | 0.250 | |
| OST ♀ x OST ♂ | | 0.01625 | | 0.11759 | | 0.138 | | 0.890 | |
| Tukey test for multiple comparisons | | | | | | | | | |
|  |  | | Estimate | | Std. Error | | z value | | Pr(>\|t\|) |
| BIR ♀ x BIR ♂ | BIR ♀ x OST ♂ | | 0.138493 | | 0.112891 | | 1.227 | | 0.609 |
|  | OST ♀ x BIR ♂ | | 0.131727 | | 0.113848 | | 1.157 | | 0.653 |
|  | OST ♀ x OST ♂ | | 0.016249 | | 0.117593 | | 0.138 | | 0.999 |
| BIR ♀ x OST ♂ | OST ♀ x BIR ♂ | | -0.006766 | | 0.101763 | | -0.066 | | 1.000 |
|  | OST ♀ x OST ♂ | | -0.122244 | | 0.105937 | | -1.154 | | 0.655 |
| OST ♀ x BIR ♂ | OST ♀ x OST ♂ | | -0.115478 | | 0.106955 | | -1.080 | | 0.701 |

n.s. *p* > 0.05, *** *p* < 0.001

Table A31: Results of the generalized linear model (number of male offspring ~ crossing, family= negative binomial) comparing the number of F2 male offspring of virgin F1 female offspring of intra- and interstrain crossings between the *L. distinguendus* strains BIR and STU and the subsequent Tukey test for multiple comparisons. The reference contained in the intercept is the crossing BIR ♀ x BIR ♂.

|  | | Estimate | | Std. Error | | z value | | Pr(>\|z\|) | |
| --- | --- | --- | --- | --- | --- | --- | --- | --- | --- |
| Intercept | | 3.0744 | | 0.1406 | | 21.872 | | <2e-16 *** | |
| BIR ♀ x STU ♂ | | 0.1361 | | 0.1940 | | 0.702 | | 0.483 | |
| STU ♀ x BIR ♂ | | 0.1465 | | 0.2029 | | 0.722 | | 0.470 | |
| STU ♀ x STU ♂ | | 0.1952 | | 0.2027 | | 0.963 | | 0.336 | |
| Tukey test for multiple comparisons | | | | | | | | | |
|  |  | | Estimate | | Std. Error | | z value | | Pr(>\|z\|) |
| BIR ♀ x BIR ♂ | BIR ♀ x STU ♂ | | 0.13613 | | 0.19397 | | 0.702 | | 0.896 |
|  | STU ♀ x BIR ♂ | | 0.14650 | | 0.20292 | | 0.722 | | 0.888 |
|  | STU ♀ x STU ♂ | | 0.19519 | | 0.20269 | | 0.963 | | 0.770 |
| BIR ♀ x STU ♂ | STU ♀ x BIR ♂ | | 0.01037 | | 0.19821 | | 0.052 | | 1.000 |
|  | STU ♀ x STU ♂ | | 0.05906 | | 0.19797 | | 0.298 | | 0.991 |
| STU ♀ x BIR ♂ | STU ♀ x STU ♂ | | 0.04870 | | 0.20675 | | 0.236 | | 0.995 |

n.s. *p* > 0.05, *** *p* < 0.001

Table A32: Results of the generalized linear model (number of male offspring ~ crossing, family= quasipoisson) comparing the number of F2 male offspring of virgin F1 female offspring of intra- and interstrain crossings between the *L. distinguendus* strains CAN and STU and the subsequent Tukey test for multiple comparisons. The reference contained in the intercept is the crossing CAN ♀ x CAN ♂.

|  | | Estimate | | Std. Error | | z value | | Pr(>\|t\|) | |
| --- | --- | --- | --- | --- | --- | --- | --- | --- | --- |
| Intercept | | 3.68492 | | 0.07845 | | 46.969 | | < 2e-16 *** | |
| CAN ♀ x STU ♂ | | 0.23653 | | 0.10495 | | 2.254 | | 0.02717 * | |
| STU ♀ x CAN ♂ | | 0.43655 | | 0.09967 | | 4.380 | | 3.85e-05 *** | |
| STU ♀ x STU ♂ | | 0.38837 | | 0.10060 | | 3.860 | | 0.00024 *** | |
| Tukey test for multiple comparisons | | | | | | | | | |
|  |  | | Estimate | | Std. Error | | z value | | Pr(>\|z\|) |
| CAN ♀ x CAN ♂ | CAN ♀ x STU ♂ | | 0.23653 | | 0.10495 | | 2.254 | | 0.109 |
|  | STU ♀ x CAN ♂ | | 0.43655 | | 0.09967 | | 4.380 | | <0.001 *** |
|  | STU ♀ x STU ♂ | | 0.38837 | | 0.10060 | | 3.860 | | <0.001 *** |
| CAN ♀ x STU ♂ | STU ♀ x CAN ♂ | | 0.20002 | | 0.09294 | | 2.152 | | 0.136 |
|  | STU ♀ x STU ♂ | | 0.15184 | | 0.09394 | | 1.616 | | 0.368 |
| STU ♀ x CAN ♂ | STU ♀ x STU ♂ | | -0.04818 | | 0.08800 | | -0.548 | | 0.947 |

n.s. *p* > 0.05, * *p* < 0.05, *** *p* < 0.001

Table A33: Results of the linear model (number of male offspring ~ crossing) comparing the number of F2 male offspring of virgin F1 female offspring of intra- and interstrain crossings between the *L. distinguendus* strains BIR and SAT and the subsequent Tukey test for multiple comparisons. The reference contained in the intercept is the crossing BIR ♀ x BIR ♂.

|  | | Estimate | | Std. Error | | t value | | Pr(>\|t\|) | |
| --- | --- | --- | --- | --- | --- | --- | --- | --- | --- |
| Intercept | | 69.105 | | 4.390 | | 15.743 | | < 2e-16 *** | |
| BIR ♀ x SAT ♂ | | -50.105 | | 14.224 | | -3.523 | | 0.000849 *** | |
| SAT ♀ x BIR ♂ | | -63.455 | | 6.130 | | -10.352 | | 1.04e-14 *** | |
| SAT ♀ x SAT ♂ | | 5.345 | | 6.130 | | 0.872 | | 0.386895 | |
| Tukey test for multiple comparisons | | | | | | | | | |
|  |  | | Estimate | | Std. Error | | t value | | Pr(>\|t\|) |
| BIR ♀ x BIR ♂ | BIR ♀ x SAT ♂ | | -50.105 | | 14.224 | | -3.523 | | 0.00399 ** |
|  | SAT ♀ x BIR ♂ | | -63.455 | | 6.130 | | -10.352 | | < 0.001 *** |
|  | SAT ♀ x SAT ♂ | | 5.345 | | 6.130 | | 0.872 | | 0.80782 |
| BIR ♀ x SAT ♂ | SAT ♀ x BIR ♂ | | -13.350 | | 14.190 | | -0.941 | | 0.76990 |
|  | SAT ♀ x SAT ♂ | | 55.450 | | 14.190 | | 3.908 | | 0.00125 ** |
| SAT ♀ x BIR ♂ | SAT ♀ x SAT ♂ | | 68.800 | | 6.051 | | 11.371 | | < 0.001 *** |

n.s. *p* > 0.05, ** *p* < 0.01, *** *p* < 0.001

Table A34: Results of the generalized linear model (number of male offspring ~ crossing, family=quasipoisson) comparing the number of F2 male offspring of virgin F1 female offspring of intra- and interstrain crossings between the *L. distinguendus* strains CAN and PFO and the subsequent Tukey test for multiple comparisons. The reference contained in the intercept is the crossing CAN ♀ x CAN ♂.

|  | | Estimate | | Std. Error | | t value | | Pr(>\|t\|) | |
| --- | --- | --- | --- | --- | --- | --- | --- | --- | --- |
| Intercept | | 3.6763 | | 0.1593 | | 23.08 | | < 2e-16 *** | |
| CAN ♀ x PFO ♂ | | N/A due to absence of mating in parental generation | | | | | | | |
| PFO ♀ x CAN ♂ | | -1.3196 | | 0.3150 | | -4.19 | | 0.000136 *** | |
| PFO ♀ x PFO ♂ | | 0.5464 | | 0.2001 | | 2.73 | | 0.009149 ** | |
| Tukey test for multiple comparisons | | | | | | | | | |
|  |  | | Estimate | | Std. Error | | z value | | Pr(>\|z\|) |
| CAN ♀ x CAN ♂ | PFO ♀ x CAN ♂ | | -1.3196 | | 0.3150 | | -4.190 | | <0.001 *** |
|  | PFO ♀ x PFO ♂ | | 0.5464 | | 0.2001 | | 2.730 | | 0.0168 * |
| PFO ♀ x CAN ♂ | PFO ♀ x PFO ♂ | | 1.8660 | | 0.2975 | | 6.271 | | <0.001 *** |

** *p* < 0.01, *** *p* < 0.001

**Male behavioural sterility**

Table A35: Comparison of the occurrence of copulation in crosses between F2 male offspring of crosses between the *L. distinguendus* strains BIR and OST and wildtype females. A Pearson's Chi-squared test was conducted for each group comparison.

|  |  | *p* value |
| --- | --- | --- |
| Group comparison BIR females | | 0.4579 n.s. |
| Group comparison OST females | | 0.8628 n.s. |

n.s. *p* > 0.05

Table A36: Comparison of the occurrence of copulation in crosses between F2 male offspring of crosses between the *L. distinguendus* strains BIR and STU and wildtype females. A 2 x 3 Fisher’s Exact test for Count Data was conducted for each group comparison

|  |  | *p* value |
| --- | --- | --- |
| Group comparison BIR females | | 0.7322 n.s. |
| Group comparison STU females | | 0.7782 n.s. |

n.s. *p* > 0.05

Table A37: Comparison of the occurrence of copulation in crosses between F2 male offspring of crosses between the *L. distinguendus* strains CAN and STU and wildtype females. A Pearson's Chi-squared test was conducted for each group comparison.

|  |  | *p* value |
| --- | --- | --- |
| Group comparison CAN females | | 0.3425 n.s. |
| Group comparison STU females | | 0.2326 n.s. |

n.s. *p* > 0.05

Table A38: Comparison of the occurrence of copulation in crosses between F2 male offspring of crosses between the *L. distinguendus* strains BIR and SAT and wildtype females. A 2 x 3 Fisher’s Exact test for Count Data was conducted for each group comparison. Single comparisons were made using the 2 x 2 Fisher’s Exact test for Count Data followed by Bonferroni corrections.

|  |  | *p* value | Significance level after Bonferroni correction (for single comparisons) |
| --- | --- | --- | --- |
| Group comparison BIR females | | 0.003076 | ** |
| BIR ♀ x (BIRxBIR) ♂ | BIR ♀ x (BIRxSAT) ♂ | 0.08754 | n.s. |
|  | BIR ♀ x (SATxBIR) ♂ | 0.006322 | * |
| BIR ♀ x (BIRxSAT) ♂ | BIR ♀ x (SATxBIR) ♂ | 0.4 | n.s. |
| Group comparison SAT females | | 0.0175 | * |
| SAT ♀ x (BIRxSAT) ♂ | SAT ♀ x (SATxBIR) ♂ | 1 | n.s. |
|  | SAT ♀ x (SATxSAT) ♂ | 0.02804 | n.s. |
| SAT ♀ x (SATxBIR) ♂ | SAT ♀ x (SATxSAT) ♂ | 0.02094 | n.s. |

n.s. *p* > 0.05, * *p* < 0.05, ** *p* < 0.01

Table A39: Comparison of the occurrence of copulation in crosses between F2 male offspring of crosses between the *L. distinguendus* strains CAN and PFO and wildtype females. A 2 x 2 Fisher’s Exact test for Count Data was conducted for each comparison

|  |  | *p* value |
| --- | --- | --- |
| CAN ♀ x (CANxCAN) ♂ | CAN ♀ x (PFOxCAN) ♂ | 0.005736 ** |
| PFO ♀ x (PFOxCAN) ♂ | PFO ♀ x (PFOxPFO) ♂ | 0.0004359 *** |

** *p* < 0.01, *** *p* < 0.001

**Male physiological sterility**

Table A40: Comparison of the occurrence of female F3 offspring in crosses between F2 male offspring of crosses between the *L. distinguendus* strains BIR and OST and wildtype females. A 2 x 3 Fisher’s Exact test for Count Data was conducted for each group comparison.

|  |  | *p* value |
| --- | --- | --- |
| Group comparison BIR females | | 1 n.s. |
| Group comparison OST females | | 1 n.s. |

n.s. *p* > 0.05

Table A41: Comparison of the occurrence of female F3 offspring in crosses between F2 male offspring of crosses between the *L. distinguendus* strains BIR and STU and wildtype females. A 2 x 3 Fisher’s Exact test for Count Data was conducted for each group comparison.

|  |  | *p* value |
| --- | --- | --- |
| Group comparison BIR females | | 1 n.s. |
| Group comparison STU females | | 0.1899 n.s. |

n.s. *p* > 0.05

Table A42: Comparison of the occurrence of female F3 offspring in crosses between F2 male offspring of crosses between the *L. distinguendus* strains CAN and STU and wildtype females. A 2 x 3 Fisher’s Exact test for Count Data was conducted for each group comparison. Single comparisons were made using the 2 x 2 Fisher’s Exact test for Count Data followed by Bonferroni corrections.

|  |  | *p* value | Significance level after Bonferroni correction (for single comparisons) |
| --- | --- | --- | --- |
| Group comparison CAN females | | 0.02944 | * |
| CAN ♀ x (CANxCAN) ♂ | CAN ♀ x (CANxSTU) ♂ | 1 | n.s. |
|  | CAN ♀ x (STUxCAN) ♂ | 0.105 | n.s. |
| CAN ♀ x (CANxSTU) ♂ | CAN ♀ x (STUxCAN) ♂ | 0.105 | n.s. |
| Group comparison STU females | | 0.7663 | n.s. |

n.s. *p* > 0.05, * *p* < 0.05

Table A43: Comparison of the occurrence of female F3 offspring in crosses between F2 male offspring of crosses between the *L. distinguendus* strains BIR and SAT and wildtype females. A 2 x 3 Fisher’s Exact test for Count Data was conducted for each group comparison. Single comparisons were made using the 2 x 2 Fisher’s Exact test for Count Data followed by Bonferroni corrections.

|  |  | *p* value | Significance level after Bonferroni correction (for single comparisons) |
| --- | --- | --- | --- |
| Group comparison BIR females | | 0.007166 | ** |
| BIR ♀ x (BIRxBIR) ♂ | BIR ♀ x (BIRxSAT) ♂ | 0.006614 | * |
|  | BIR ♀ x (SATxBIR) ♂ | 0.03021 | n.s. |
| BIR ♀ x (BIRxSAT) ♂ | BIR ♀ x (SATxBIR) ♂ | 0.674 | n.s. |
| Group comparison SAT females | | 0.2745 | n.s. |

n.s. *p* > 0.05, * *p* < 0.05, ** *p* < 0.01

Table A44: Comparison of the occurrence of female F3 offspring in crosses between F2 male offspring of crosses between the *L. distinguendus* strains CAN and PFO and wildtype females. Comparisons were made using the 2 x 2 Fisher’s Exact test for Count Data.

|  |  | *p* value |
| --- | --- | --- |
| CAN ♀ x (CANxCAN) ♂ | CAN ♀ x (PFOxCAN) ♂ | 8.446e-05 *** |
| PFO ♀ x (PFOxCAN) ♂ | PFO ♀ x (PFOxPFO) ♂ | 0.008316 ** |

** *p* < 0.01, *** *p* < 0.001

**Male physiological reduced fertility**

Table A45: Results of the linear model (number of female offspring ~ crossing) comparing the number of F3 female offspring of F2 male offspring of intra- and interstrain crossings between the *L. distinguendus* strains BIR and OST and wildtype females and the subsequent Tukey test for multiple comparisons. The reference contained in the intercept is the crossing BIR ♀ x (BIRxBIR) ♂.

|  | | Estimate | Std. Error | | t value | | | Pr(>\|t\|) | |
| --- | --- | --- | --- | --- | --- | --- | --- | --- | --- |
| Intercept | | 38.056 | 4.568 | | 8.331 | | | 3.1e-13 *** | |
| BIR ♀ x (BIRxOST) ♂ | | -3.898 | 6.375 | | -0.611 | | | 0.542 | |
| BIR ♀ x (OSTxBIR) ♂ | | -7.213 | 6.375 | | -1.132 | | | 0.260 | |
| OST ♀ x (BIRxOST) ♂ | | -2.266 | 6.375 | | -0.355 | | | 0.723 | |
| OST ♀ x (OSTxBIR) ♂ | | -1.371 | 6.375 | | -0.215 | | | 0.830 | |
| OST ♀ x (OSTxOST) ♂ | | -1.056 | 6.460 | | -0.163 | | | 0.871 | |
| Tukey test for multiple comparisons | | | | | | | | | |
|  |  | | | Estimate | | Std. Error | z value | | Pr(>\|t\|) |
| BIR ♀ x (BIRxBIR) ♂ | BIR ♀ x (BIRxOST) ♂ | | | -3.8977 | | 6.3747 | -0.611 | | 0.990 |
|  | BIR ♀ x (OSTxBIR) ♂ | | | -7.2135 | | 6.3747 | -1.132 | | 0.867 |
|  | OST ♀ x (BIRxOST) ♂ | | | -2.2661 | | 6.3747 | -0.355 | | 0.999 |
|  | OST ♀ x (OSTxBIR) ♂ | | | -1.3713 | | 6.3747 | -0.215 | | 1.000 |
|  | OST ♀ x (OSTxOST) ♂ | | | -1.0556 | | 6.4603 | -0.163 | | 1.000 |
| BIR ♀ x (BIRxOST) ♂ | BIR ♀ x (OSTxBIR) ♂ | | | -3.3158 | | 6.2880 | -0.527 | | 0.995 |
|  | OST ♀ x (BIRxOST) ♂ | | | 1.6316 | | 6.2880 | 0.259 | | 1.000 |
|  | OST ♀ x (OSTxBIR) ♂ | | | 2.5263 | | 6.2880 | 0.402 | | 0.999 |
|  | OST ♀ x (OSTxOST) ♂ | | | 2.8421 | | 6.3747 | 0.446 | | 0.998 |
| BIR ♀ x (OSTxBIR) ♂ | OST ♀ x (BIRxOST) ♂ | | | 4.9474 | | 6.2880 | 0.787 | | 0.969 |
|  | OST ♀ x (OSTxBIR) ♂ | | | 5.8421 | | 6.2880 | 0.929 | | 0.938 |
|  | OST ♀ x (OSTxOST) ♂ | | | 6.1579 | | 6.3747 | 0.966 | | 0.928 |
| OST ♀ x (BIRxOST) ♂ | OST ♀ x (OSTxBIR) ♂ | | | 0.8947 | | 6.2880 | 0.142 | | 1.000 |
|  | OST ♀ x (OSTxOST) ♂ | | | 1.2105 | | 6.3747 | 0.190 | | 1.000 |
| OST ♀ x (OSTxBIR) ♂ | OST ♀ x (OSTxOST) ♂ | | | 0.3158 | | 6.3747 | 0.050 | | 1.000 |

n.s. *p* > 0.05, *** *p* < 0.001

Table A46: Results of the generalized linear model (number of female offspring ~ crossing, family= negative binomial) comparing the number of F3 female offspring of F2 male offspring of intra- and interstrain crossings between the *L. distinguendus* strains BIR and STU and wildtype females and the subsequent Tukey test for multiple comparisons. The reference contained in the intercept is the crossing BIR ♀ x (BIRxBIR) ♂.

|  | | Estimate | Std. Error | | z value | | | Pr(>\|z\|) | |
| --- | --- | --- | --- | --- | --- | --- | --- | --- | --- |
| Intercept | | 2.2029 | 0.1082 | | 20.359 | | | < 2e-16 *** | |
| BIR ♀ x (BIRxSTU) ♂ | | 0.1205 | 0.1530 | | 0.788 | | | 0.43075 | |
| BIR ♀ x (STUxBIR) ♂ | | 0.1597 | 0.1808 | | 0.884 | | | 0.37685 | |
| STU ♀ x (BIRxSTU) ♂ | | 0.1988 | 0.1729 | | 1.149 | | | 0.25038 | |
| STU ♀ x (STUxBIR) ♂ | | 0.4221 | 0.1480 | | 2.851 | | | 0.00435 ** | |
| STU ♀ x (STUxSTU) ♂ | | 0.2240 | 0.1531 | | 1.463 | | | 0.14345 | |
| Tukey test for multiple comparisons | | | | | | | | | |
|  |  | | | Estimate | | Std. Error | z value | | Pr(>\|z\|) |
| BIR ♀ x (BIRxBIR) ♂ | BIR ♀ x (BIRxSTU) ♂ | | | 0.12051 | | 0.15295 | 0.788 | | 0.969 |
|  | BIR ♀ x (STUxBIR) ♂ | | | 0.15974 | | 0.18076 | 0.884 | | 0.950 |
|  | STU ♀ x (BIRxSTU) ♂ | | | 0.19877 | | 0.17293 | 1.149 | | 0.859 |
|  | STU ♀ x (STUxBIR) ♂ | | | 0.42212 | | 0.14805 | 2.851 | | 0.049 * |
|  | STU ♀ x (STUxSTU) ♂ | | | 0.22405 | | 0.15313 | 1.463 | | 0.685 |
| BIR ♀ x (BIRxSTU) ♂ | BIR ♀ x (STUxBIR) ♂ | | | 0.03923 | | 0.18070 | 0.217 | | 1.000 |
|  | STU ♀ x (BIRxSTU) ♂ | | | 0.07826 | | 0.17287 | 0.453 | | 0.998 |
|  | STU ♀ x (STUxBIR) ♂ | | | 0.30160 | | 0.14797 | 2.038 | | 0.318 |
|  | STU ♀ x (STUxSTU) ♂ | | | 0.10353 | | 0.15306 | 0.676 | | 0.984 |
| BIR ♀ x (STUxBIR) ♂ | STU ♀ x (BIRxSTU) ♂ | | | 0.03903 | | 0.19790 | 0.197 | | 1.000 |
|  | STU ♀ x (STUxBIR) ♂ | | | 0.26237 | | 0.17657 | 1.486 | | 0.671 |
|  | STU ♀ x (STUxSTU) ♂ | | | 0.06430 | | 0.18086 | 0.356 | | 0.999 |
| STU ♀ x (BIRxSTU) ♂ | STU ♀ x (STUxBIR) ♂ | | | 0.22335 | | 0.16854 | 1.325 | | 0.769 |
|  | STU ♀ x (STUxSTU) ♂ | | | 0.02528 | | 0.17303 | 0.146 | | 1.000 |
| STU ♀ x (STUxBIR) ♂ | STU ♀ x (STUxSTU) ♂ | | | -0.19807 | | 0.14816 | -1.337 | | 0.762 |

n.s. *p* > 0.05, * *p* < 0.05, ** *p* < 0.01, *** *p* < 0.001

Table A47: Results of the generalized linear model (number of female offspring ~ crossing, family= quasipoisson) comparing the number of F3 female offspring of F2 male offspring of intra- and interstrain crossings between the *L. distinguendus* strains CAN and STU and wildtype females and the subsequent Tukey test for multiple comparisons. The reference contained in the intercept is the crossing CAN ♀ x (CANxCAN) ♂.

|  | Estimate | Std. Error | | z value | | | Pr(>\|t\|) | |
| --- | --- | --- | --- | --- | --- | --- | --- | --- |
| Intercept | 3.37092 | 0.11436 | | 29.477 | | | < 2e-16 *** | |
| CAN ♀ x (CANxSTU) ♂ | -0.87728 | 0.21100 | | -4.158 | | | 6.89e-05 *** | |
| CAN ♀ x (STUxCAN) ♂ | -1.02272 | 0.24319 | | -4.205 | | | 5.77e-05 *** | |
| STU ♀ x (CANxSTU) ♂ | -0.51190 | 0.19003 | | -2.694 | | | 0.00831 ** | |
| STU ♀ x (STUxCAN) ♂ | -0.26660 | 0.17933 | | -1.487 | | | 0.14033 | |
| STU ♀ x (STUxSTU) ♂ | 0.08105 | 0.16553 | | 0.490 | | | 0.62547 | |
| Tukey test for multiple comparisons | | | | | | | | |
|  |  | | Estimate | | Std. Error | z value | | Pr(>\|z\|) |
| CAN ♀ x (CANxCAN) ♂ | CAN ♀ x (CANxSTU) ♂ | | -0.87728 | | 0.21100 | -4.158 | | <0.001 *** |
|  | CAN ♀ x (STUxCAN) ♂ | | -1.02272 | | 0.24319 | -4.205 | | <0.001 *** |
|  | STU ♀ x (CANxSTU) ♂ | | -0.51190 | | 0.19003 | -2.694 | | 0.0737 |
|  | STU ♀ x (STUxCAN) ♂ | | -0.26660 | | 0.17933 | -1.487 | | 0.6657 |
|  | STU ♀ x (STUxSTU) ♂ | | 0.08105 | | 0.16553 | 0.490 | | 0.9964 |
| CAN ♀ x (CANxSTU) ♂ | CAN ♀ x (STUxCAN) ♂ | | -0.14544 | | 0.27840 | -0.522 | | 0.9951 |
|  | STU ♀ x (CANxSTU) ♂ | | 0.36538 | | 0.23340 | 1.565 | | 0.6137 |
|  | STU ♀ x (STUxCAN) ♂ | | 0.61068 | | 0.22478 | 2.717 | | 0.0694 |
|  | STU ♀ x (STUxSTU) ♂ | | 0.95833 | | 0.21393 | 4.480 | | <0.001 *** |
| CAN ♀ x (STUxCAN) ♂ | STU ♀ x (CANxSTU) ♂ | | 0.51083 | | 0.26286 | 1.943 | | 0.3676 |
|  | STU ♀ x (STUxCAN) ♂ | | 0.75613 | | 0.25524 | 2.962 | | 0.0350 * |
|  | STU ♀ x (STUxSTU) ♂ | | 1.10377 | | 0.24573 | 4.492 | | <0.001 *** |
| STU ♀ x (CANxSTU) ♂ | STU ♀ x (STUxCAN) ♂ | | 0.24530 | | 0.20522 | 1.195 | | 0.8348 |
|  | STU ♀ x (STUxSTU) ♂ | | 0.59295 | | 0.19327 | 3.068 | | 0.0255 * |
| STU ♀ x (STUxCAN) ♂ | STU ♀ x (STUxSTU) ♂ | | 0.34765 | | 0.18276 | 1.902 | | 0.3926 |

n.s. *p* > 0.05, * *p* < 0.05, ** *p* < 0.01, *** *p* < 0.001

Table A48: Results of the linear model (number of female offspring ~ crossing) comparing the number of F3 female offspring of F2 male offspring of intra- and interstrain crossings between the *L. distinguendus* strains BIR and SAT and wildtype females and the subsequent Tukey test for multiple comparisons. The reference contained in the intercept is the crossing BIR ♀ x (BIRxBIR) ♂.

|  | Estimate | Std. Error | | t value | | | Pr(>\|t\|) | |
| --- | --- | --- | --- | --- | --- | --- | --- | --- |
| Intercept | 55.583 | 5.571 | | 9.978 | | | 1.39e-13 *** | |
| BIR ♀ x (BIRxSAT) ♂ | -34.583 | 12.457 | | -2.776 | | | 0.00767 ** | |
| BIR ♀ x (SATxBIR) ♂ | -32.583 | 9.649 | | -3.377 | | | 0.00141 ** | |
| SAT ♀ x (BIRxSAT) ♂ | -27.333 | 8.808 | | -3.103 | | | 0.00312 ** | |
| SAT ♀ x (SATxBIR) ♂ | 6.340 | 7.725 | | 0.821 | | | 0.41567 | |
| SAT ♀ x (SATxSAT) ♂ | 1.283 | 7.474 | | 0.172 | | | 0.86435 | |
| Tukey test for multiple comparisons | | | | | | | | |
|  |  | | Estimate | | Std. Error | t value | | Pr(>\|t\|) |
| BIR ♀ x (BIRxBIR) ♂ | BIR ♀ x (BIRxSAT) ♂ | | -34.583 | | 12.457 | -2.776 | | 0.07405 |
|  | BIR ♀ x (SATxBIR) ♂ | | -32.583 | | 9.649 | -3.377 | | 0.01578 * |
|  | SAT ♀ x (BIRxSAT) ♂ | | -27.333 | | 8.808 | -3.103 | | 0.03291 * |
|  | SAT ♀ x (SATxBIR) ♂ | | 6.340 | | 7.725 | 0.821 | | 0.96040 |
|  | SAT ♀ x (SATxSAT) ♂ | | 1.283 | | 7.474 | 0.172 | | 0.99998 |
| BIR ♀ x (BIRxSAT) ♂ | BIR ♀ x (SATxBIR) ♂ | | 2.000 | | 13.646 | 0.147 | | 0.99999 |
|  | SAT ♀ x (BIRxSAT) ♂ | | 7.250 | | 13.065 | 0.555 | | 0.99304 |
|  | SAT ♀ x (SATxBIR) ♂ | | 40.923 | | 12.361 | 3.311 | | 0.01899 * |
|  | SAT ♀ x (SATxSAT) ♂ | | 35.867 | | 12.205 | 2.939 | | 0.04982 * |
| BIR ♀ x (SATxBIR) ♂ | SAT ♀ x (BIRxSAT) ♂ | | 5.250 | | 10.422 | 0.504 | | 0.99556 |
|  | SAT ♀ x (SATxBIR) ♂ | | 38.923 | | 9.524 | 4.087 | | 0.00190 ** |
|  | SAT ♀ x (SATxSAT) ♂ | | 33.867 | | 9.322 | 3.633 | | 0.00757 ** |
| SAT ♀ x (BIRxSAT) ♂ | SAT ♀ x (SATxBIR) ♂ | | 33.673 | | 8.672 | 3.883 | | 0.00358 ** |
|  | SAT ♀ x (SATxSAT) ♂ | | 28.617 | | 8.449 | 3.387 | | 0.01533 * |
| SAT ♀ x (SATxBIR) ♂ | SAT ♀ x (SATxSAT) ♂ | | -5.056 | | 7.313 | -0.691 | | 0.98112 |

n.s. *p* > 0.05, * *p* < 0.05, ** *p* < 0.01, *** *p* < 0.001

Table A49: Results of the linear model (number of female offspring ~ crossing) comparing the number of F3 female offspring of F2 male offspring of intra- and interstrain crossings between the *L. distinguendus* strains CAN and PFO and wildtype females and the subsequent Tukey test for multiple comparisons. The reference contained in the intercept is the crossing CAN ♀ x (CANxCAN) ♂.

|  | Estimate | Std. Error | | t value | | | Pr(>\|t\|) | |
| --- | --- | --- | --- | --- | --- | --- | --- | --- |
| Intercept | 45.52632 | 4.98624 | | 9.130 | | | 1.55e-12 *** | |
| CAN ♀ x (PFOxCAN) ♂ | -21.02632 | 10.17812 | | -2.066 | | | 0.0437 * | |
| PFO ♀ x (PFOxCAN) ♂ | -20.52632 | 7.82306 | | -2.624 | | | 0.0113 * | |
| PFO ♀ x (PFOxPFO) ♂ | 0.07368 | 6.96291 | | 0.011 | | | 0.9916 | |
| Tukey test for multiple comparisons | | | | | | | | |
|  |  | | Estimate | | Std. Error | t value | | Pr(>\|t\|) |
| CAN ♀ x (CANxCAN) ♂ | CAN ♀ x (PFOxCAN) ♂ | | -21.02632 | | 10.17812 | -2.066 | | 0.1730 |
|  | PFO ♀ x (PFOxCAN) ♂ | | -20.52632 | | 7.82306 | -2.624 | | 0.0513 |
|  | PFO ♀ x (PFOxPFO) ♂ | | 0.07368 | | 6.96291 | 0.011 | | 1.0000 |
| CAN ♀ x (PFOxCAN) ♂ | PFO ♀ x (PFOxCAN) ♂ | | 0.50000 | | 10.72703 | 0.047 | | 1.0000 |
|  | PFO ♀ x (PFOxPFO) ♂ | | 21.10000 | | 10.11687 | 2.086 | | 0.1666 |
| PFO ♀ x (PFOxCAN) ♂ | PFO ♀ x (PFOxPFO) ♂ | | -20.60000 | | 7.74320 | -2.660 | | 0.0469 * |

n.s. *p* > 0.05, * *p* < 0.05, *** *p* < 0.001

**Cytoplasmic incompatibility**

Table A50: Comparison of the occurrence of F1 female offspring in crosses between females and males of the *Spiroplasma*-carrying STU(+) and potentially endosymbiont-infected BIR(+) strains using a 2 x 4 Fisher’s Exact Test for Count Data for the group comparison and 2 x 2 Fisher’s Exact Test for Count Data followed by Bonferroni correction for single comparisons.

|  |  | | *p* value | Significance level after Bonferroni correction |
| --- | --- | --- | --- | --- |
| Group comparison | | | 1.197e-06 | *** |
| BIR(+) ♀ x BIR(+) ♂ | | BIR(+) ♀ x STU(+) | 1.193e-05 | *** |
|  | | STU(+) ♀ x BIR(+) ♂ | 0.1498 | n.s. |
|  | | STU(+)♀x STU(+)♂ | 0.1483 | n.s. |
| BIR(+) ♀ x STU(+) | | STU(+) ♀ x BIR(+) ♂ | 0.0002763 | ** |
|  | | STU(+)♀x STU(+)♂ | 0.0004133 | ** |
| STU(+) ♀ x BIR(+) ♂ | | STU(+)♀x STU(+)♂ | 1 | n.s. |

n.s. *p* > 0.05, ** *p* < 0.01, *** *p* < 0.001

Table A51: Comparison of the occurrence of F1 female offspring in crosses between untreated females and males of the strains STU(+) and BIR(+) strains in the combinations STU females x STU males, STU females x BIR males, BIR females x BIR males and untreated BIR(+) females with tetracycline-treated STU(-) males using a 2 x 4 Fisher’s Exact Test for Count Data for the group comparison.

|  |  | | *p* value | Significance level after Bonferroni correction |
| --- | --- | --- | --- | --- |
| Group comparison | | | 0.02489 | * |
| BIR(+) ♀ x BIR(+) ♂ | | BIR(+) ♀ x STUtet(-) ♂ | 1 | n.s. |
|  | | STU(+) ♀ x BIR(+) ♂ | 0.1498 | n.s. |
|  | | STU(+)♀x STU(+)♂ | 0.1483 | n.s. |
| BIR(+) ♀ x STUtet(-) ♂ | | STU(+) ♀ x BIR(+) ♂ | 0.0254 | n.s. |
|  | | STU(+)♀x STU(+)♂ | 0.03977 | n.s. |
| STU(+) ♀ x BIR(+) ♂ | | STU(+)♀x STU(+)♂ | 1 | n.s. |

n.s. *p* > 0.05, * *p* < 0.05

**Sperm count**

Table A52: Statistical analyses of sperm counts in seminal vesicles and spermathecae of F2 males and females crossed to F2 males in crosses between CAN and STU females and males.

|  | Statistics |
| --- | --- |
| Seminal vesicles |  |
| Parental cross of tested F2 males |  |
| CAN ♀ x CAN ♂ | *p*  = 3.821e-08  *W* = 7251  Wilcoxon ranked sum test |
| STU ♀ x CAN ♂ |  |
| CAN ♀ x STU ♂ | *p* = 0.5808  *W* = 5226.5  Wilcoxon ranked sum test |
| STU ♀ x STU ♂ |  |
| Spermathecae |  |
| Tested females x parental cross of tested F2 males |  |
| CAN ♀ x (CAN ♀ x CAN ♂) ♂ | n.s.  *p*  = 0.1365  *W* = 660.5  Wilcoxon ranked sum test |
| CAN ♀ x (STU ♀ x CAN ♂) ♂ |  |
| STU ♀ x (CAN ♀ x STU ♂) ♂ | n.s.  *p* = 0.4026  *t*  = -0.84294  Welch two sample t-test |
| STU ♀ x (STU ♀ x STU ♂) ♂ |  |
